# Preliminary Phytochemical and Biological Evaluation of *Rudbeckia hirta* Flowers

**DOI:** 10.3390/plants12152871

**Published:** 2023-08-04

**Authors:** Ana Flavia Burlec, Łukasz Pecio, Cornelia Mircea, Cristina Tuchiluș, Andreia Corciovă, Corina Danciu, Oana Cioancă, Ioana Cezara Caba, Solomiia Pecio, Wiesław Oleszek, Monica Hăncianu

**Affiliations:** 1 Faculty of Pharmacy, “Grigore T. Popa” University of Medicine and Pharmacy, 16 University Street, 700115 Iasi, Romania; ana-flavia.l.burlec@umfiasi.ro (A.F.B.); oana.cioanca@umfiasi.ro (O.C.); ioana-cezara.caba@umfiasi.ro (I.C.C.); mhancianu@yahoo.com (M.H.); 2 Department of Biochemistry and Crop Quality, Institute of Soil Science and Plant Cultivation—State Research Institute, Czartoryskich 8 Street, 24-100 Puławy, Poland; lpecio@iung.pulawy.pl (Ł.P.); skozachok@iung.pulawy.pl (S.P.); wieslaw.oleszek@iung.pulawy.pl (W.O.); 3 Department of Chemistry of Natural Products, Medical University of Lublin, 1 Chodźki Street, 20-093 Lublin, Poland; 4 Faculty of Medicine, “Grigore T. Popa” University of Medicine and Pharmacy, 16 University Street, 700115 Iasi, Romania; cristina.tuchilus@umfiasi.ro; 5 Department of Pharmacognosy, University of Medicine and Pharmacy “Victor Babes”, Eftimie Murgu Square, No. 2, 300041 Timisoara, Romania; corina.danciu@umft.ro

**Keywords:** black-eyed Susan, methanolic extract, UHPLC-HR-MS, sesquiterpenoids, phenolics, fatty acids, antioxidant activity, antimicrobial activity, MCF-7 cell line

## Abstract

Black-eyed Susan (*Rudbeckia hirta* L.), a flowering plant with various traditional medicinal uses, has recently garnered interest for its therapeutic properties. However, little is known about the potential therapeutic activities of the plant species. The current study focused on conducting a comprehensive investigation into the chemical composition and bioactivity of black-eyed Susan cultivated in Romania. Untargeted metabolite profiling and UHPLC-HR-MS phytochemical analysis of the studied extract revealed the presence of more than 250 compounds pertaining to different classes, including sesquiterpene lactones, polyphenolic acids, flavonoids, amino acids, and fatty acids. The tested extract exhibited inhibitory activity against Gram-positive bacteria and showed promising antifungal activity. It also demonstrated potent antioxidant properties through iron chelation and 15-LOX inhibition capacities, as well as inhibition of cell growth, particularly on the MCF-7 cell line, suggesting potential anticancer effects. Therefore, current research provides valuable information on the antioxidant, antimicrobial, and antitumor potential of *Rudbeckia hirta* flowers. Implicitly, the discovery of such a wide range of biosubstances, together with the biological activity observed for the studied extract in these preliminary in vitro studies, paves the way for future investigation of the potential application of the plant in the pharmaceutical and nutraceutical sectors.

## 1. Introduction

The genus Rudbeckia contains more than 20 species, of which *Rudbeckia hirta* L., *R. fulgida*, *R. laciniata*, *R. maxima*, *R. occidentalis* and *R. triloba* are best known for their decorative value [1,2]. Carl Linnaeus named this genus in honor of Olaf Rudbeck, the son, who was a botanist and professor at Uppsala University and whose father founded Sweden’s first botanical garden [3].

*R. hirta*, also known as black-eyed Susan or yellow coneflower, is a popular wildflower native to North America. The plant is known for its striking yellow petals and dark center that resembles a black eye, giving it its common name. A hardy and easy-to-grow plant, *R. hirta* is often seen in meadows and prairies but also along roadsides [4]. The plant can bloom well into the fall but usually does not bloom until mid to late summer. Its drought tolerance makes it a great option for gardeners looking for low-maintenance plants. The species belongs to the Asteraceae family, which is the best-represented and most widespread family of dicotyledonous plants and consists of approximately 1500 genera and more than 23,000 species that illustrate a great morphological diversity [5,6].

*R. hirta* has been classified as annual, biennial or perennial, depending on its habitat and genetic characteristics [7]. The plant is a herbaceous species with a semi-erect stem that is covered with bristles and can grow up to 1 m in height. The stem supports a single flower head and is surrounded by a basal rosette of leaves. Small, dark brown or black disk florets form the dark core of the flower, which is surrounded by a ring of yellow ray florets [8]. Like the stem, the leaves and bracts are covered with hairs, which explains the scientific name of the species, the Latin term “*hirta*” meaning “hairy” [9].

The plant is commonly used for landscaping but can also be used to control soil erosion and provide shelter for several species of birds. In addition to its ornamental value, the species has long been used in traditional medicine in several regions of North America. For example, the Cherokee and Iroquois tribes used the plant as an anthelmintic, especially in the form of infusions prepared from the roots [10]. Various root extracts have also been used to treat gynecological conditions and sexually transmitted diseases. The plant has been used to treat fevers, colds, and headaches and is believed to have anti-inflammatory and analgesic properties [11]. In addition, the plant’s roots, leaves, and flowers have been used to make tea and various extracts, as well as poultices and ointments. The plant is also thought to have antimicrobial properties [12,13]. Given these potential applications, the plant becomes attractive for additional purification processes that may lead to the discovery of therapeutically useful compounds.

Given that *R. hirta* can be found in different geographical regions, its growth and development, as well as the production of secondary metabolites, can be influenced by various factors such as origin, the altitude at which the plant grows, amount of rainfall, temperature, exposure to sunlight, soil quality and composition. In addition, the number of secondary metabolites produced by the plant can vary with the age and degree of growth, but also with the conditions of collection, drying and storage of the plant material [14].

The species has a number of chemical constituents that are thought to contribute to its potential bioactive properties. These include flavonoids with antioxidant, anti-inflammatory and antibacterial activities, such as quercetin and its glycosides, kaempferol and quercetagetin derivatives, patuletin and eupatolin. Several polyphenolic acids, such as caffeic and chlorogenic acids, are also known to be present in the plant. Other chemical constituents of *R. hirta* include terpenes, which are thought to be responsible for the plant’s distinctive taste and smell and its antibacterial properties. The inflorescences and roots of the species also contain several classes of polyacetylenic and polyinic compounds, such as thiophenes, thiarubrines and tridecapentaynenes, with implications for the insecticidal properties observed in different extracts obtained from the plant [15,16]. Sesquiterpenes, representing the Asteraceae family, i.e., germacrene D, δ-cadinene and a pseudoguaianolide-type lactone—rudbeckolide, which showed potent 5-LOX inhibitory activity in vitro, were also isolated from the flowers of *R. hirta* [17,18].

Considering that the species is generally associated with its ornamental role, very few studies have had as focus the investigation of the secondary metabolites found in the plant and their possible therapeutic use. This is the first report aimed at the detailed phytochemical characterization of the specific metabolites derived from the methanolic extract of *R. hirta* flowers using LC-HRMS analysis and molecular networks (MetGem) for the identification and visualization of numerous groups of plant metabolites. The obtained extract was tested in vitro for antioxidant, antibacterial and antifungal effects, and cytotoxicity evaluation against different cancer cell lines was also performed.

## 2. Results

### 2.1. Identification of Compounds Found in the Extract

**Figure 1:**
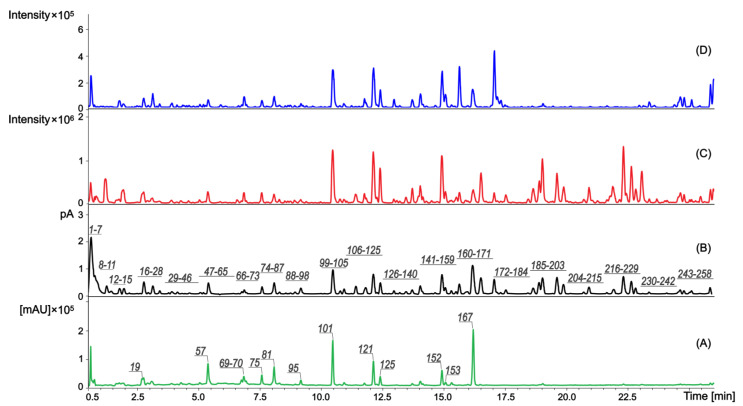
UHPLC-HRESIMS-PDA-CAD profiles of the *R. hirta* extract—PDA 200–800 nm (**A**); CAD (**B**); ESI(+) (**C**); ESI(−) (**D**). The numbers above the peaks refer to the designated metabolites **1**–**258** presented in Table S1.

HRMS (high-resolution mass spectrometry) analysis of the complex matrix of *R. hirta* flower methanolic extract detected 258 plant metabolites (Figure 1). Of these, 248 were tentatively identified using MS-DIAL [19,20] and public databases. In addition, MS-FINDER [20] and SIRIUS [21] were used for in silico fragmentation analysis. We also used MetGem software [22] to generate molecular networks (MNs) similar to those generated by Global Natural Products Social (GNPS) [23,24] based on the observation that compounds with a high degree of chemical similarity have comparable MS/MS fragments.

**Figure 2:**
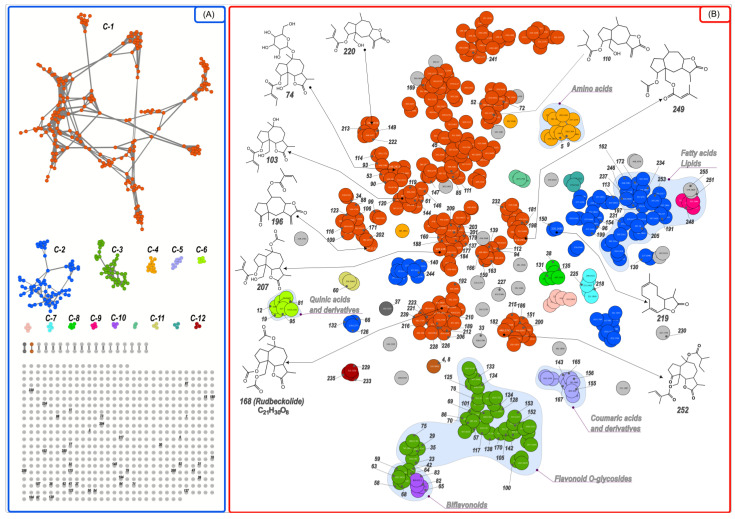
Molecular networks (**A**) and t-SNE (**B**) outputs in the positive mode ESI(+) for the *R. hirta* UHPLC-HRESIMS data. The designations C-1 through C-12 correspond to clusters (+MetGem Cluster) of compounds with similar chemical properties. Detailed data are included in Table S1 [17,25,26,28,29,30,31,32,33,34,35,36,37,38,39,40,41,42,43,44,45,46,47,48,49,50,51,52,53,54,55,56,57,58,59].

**Figure 3:**
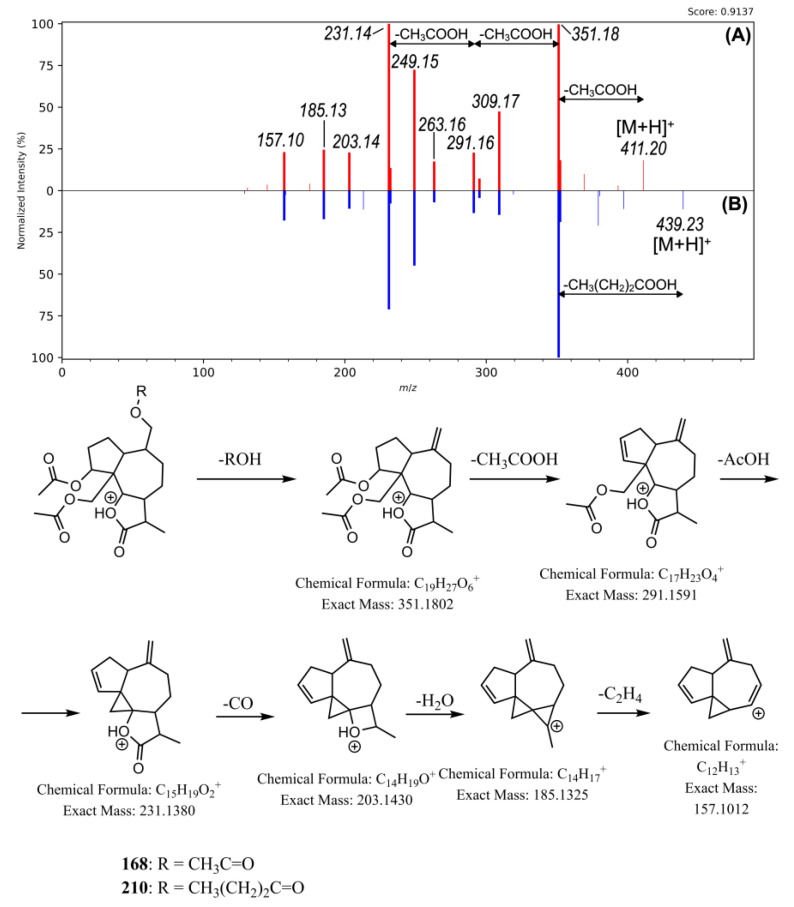
Comparison of the MS/MS spectra of [M + NH_4_]^+^ ions of compound **168** (**A**) and compound **210** (**B**) showing a strong cosine score (0.9137) in the C-1 cluster of molecular network in positive mode.

The main class of the identified specific metabolites belonged to sesquiterpenoids, which comprised 84 compounds and accounted for 29.7% of the total CAD (charged aerosol detector) peak area (Tables S1 and S2). The major group of this class consisting of 65 metabolites, were sesquiterpene lactones (accounting for 26.0% of the total CAD peak area), mainly ascribing to the cluster C-1 formed in MN in the positive ionization mode (Figure 2A). Pseudoguaianolide-type sesquiterpene lactones such as rudbeckin A (**45**) [25], rudbeckolide (**168**) [17], rudmollin (**114**), acetoxyrudmollin (**52**, **72**, **149**) [26,27], previously isolated from the genus *Rudbeckia* L., were tentatively identified in the studied extract. The MS/MS spectra of these compounds are difficult to decipher in the absence of standards. Molecular networks, and in particular nonlinear multivariate embedding methods such as the t-SNE (t-distributed Stochastic Neighborhood Embedding) algorithm available in MetGem, make such analysis somewhat easier. It is important to note that since the spatial orientation of the MS/MS network is randomly generated when the network is rendered by MetGem (Figure 2A), the location of the nodes within the planar representation of the MS/MS network is not related to the nature of the molecule. On the other hand, in the t-SNE output (Figure 2B), patterns can be easily distinguished by simple visual analysis and in an unsupervised manner, as this algorithm tends to preserve local distances and distort global distances, i.e., if two clusters are close in original space, they have a statistically greater chance of being close in the t-SNE projection than distant clusters. As a result, certain subfamilies can be easily distinguished in t-SNE. One such subfamily includes the triacetylated compound, rudbeckolide (**168**), in addition to a number of other triacylated rudbeckin A derivatives—typically containing two acetyl groups and a propionyl (**189**), a butyryl (**210**, **212**), a butenoyl (**206**), a methylbutanoyl (**226**, **228**), and a methylbutenoyl (**221**, **223**) groups, but also one acetyl, methylbutenoyl and propionyl group each (**239**). Figure 3 shows a comparison of MS/MS spectra of the putative rudbeckolide (**168**) with diacetyl-butyryl-rudbeckin A (**210**) found in the C-1 cluster of the MN in positive mode ESI(+), along with the fragmentation pathway characteristic of both compounds. Subfamily adjacent to the previous one is the one containing diacylated derivatives of rudbeckin A—containing two acetyl substituents (**137**), one acetyl and one propionyl (**160**), one acetyl and one butyryl (**177**, **179**, **188**), one acetyl and one methylbutanoyl (**201**, **203**, **209**), one acetyl and one methylbutenoyl (**184**, **207**). A subfamily of diacylated dehydrorudbeckin A (**151**, **182**, **186**, **200**, **215**, **252**) and dihydrorudmollin derivatives (**181**, **198**, **232**, **249**) can also be distinguished. These, in turn, are adjacent to monoacylated derivatives of rudbeckin A (**61**, **103**, **119**, **120**, **144**), confertin (**196**, **202**), or rudmollin (**149**, **213**, **220**, **222**). Several glycosylated sesquiterpene derivatives were also observed (**53**, **74**, **88**, **90**, **93**). MS/MS spectra, of course, do not carry adequate structural or stereochemical information. Thus, it should be noted that although the subfamilies seen in the t-SNE outputs group compounds with similar acylation patterns and sesquiterpene cores, they may also contain different cores from those proposed.

**Figure 4:**
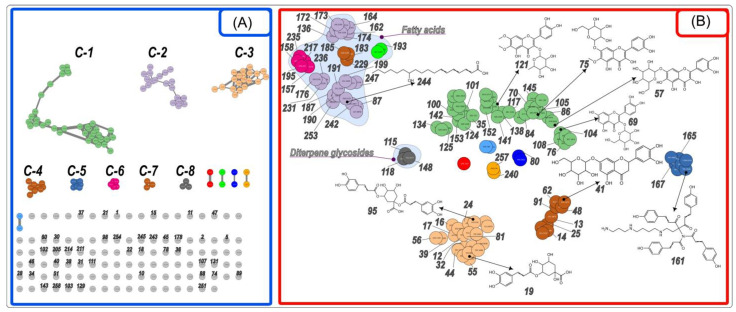
Molecular networks (**A**) and t-SNE (**B**) outputs in the negative mode ESI(−) for the *R. hirta* UHPLC-HRESIMS data. The designations C-1 through C-8 correspond to clusters (-MetGem Cluster) of compounds with similar chemical properties. Detailed data are included in Table S1.

A total of 11 metabolites belonging to the chlorogenic acid group (esters with quinic acid and certain cinnamic acids) comprising 6.7% of the total CAD peak area were tentatively identified (Table S1) and presented in cluster C-3 formed in MN in negative ionization mode (Figure 4A). A deprotonated ion of C_16_H_18_O_9_ isomers was observed at *m*/*z* 353, giving the following fragmentation ions at *m*/*z* 191, *m*/*z* 179, *m*/*z* 173, *m*/*z* 135, and *m*/*z* 127. These compounds were distinguished as 3-*O*-caffeoylquinnic acid (3-CQA) (**12**), 5-CQA (**19**), 4-CQA (**24**) according to the base peak, the intensity of the product ions, the literature data, library hit matching and elution principal rules [41,43,60]. The identified 5-*O*-feruloylquinic acid (FQA) (**44**) gave the deprotonated ion at *m*/*z* 367 (C_17_H_19_O_9_)^−^ and released the fragment ions at *m*/*z* 193, *m*/*z* 191 (base peak). The series of *p*-coumaroylquinic acids (*p*CoQA) [*m*/*z* 337 (C₁₆H₁₈O₈)^−^] were also detected. *Trans-*3-*p*CoQA (**17**) eluted at 2.6 min and gave the base beak at *m*/*z* 163; its stereoisomers *cis*-3-*p*CoQA (**16**) eluted slightly earlier at 2.5 min. *Trans*-5-*p*CoQA (**32**) is less hydrophobic than *cis*-5-*p*CoQA (**55**). Both molecules released the most abundant fragment ion at *m*/*z* 191. *Trans*-4-*p*CoQA (**36**) gave the base peak at *m*/*z* 173.

Two dicaffeoylquinic acid (diCQA) isomers were identified by their precursor ion at *m*/*z* 515 (C_25_H_23_O_12_)^−^ and released product ions at *m*/*z* 353, *m*/*z* 191, *m*/*z* 179, *m*/*z* 173, *m*/*z* 135. Compounds **81** and **95** produced major product ions at *m*/*z* 191 and *m*/*z* 173, respectively, which were considered as 1,3-diCQA and 1,5-diCQA according to the literature [42,43]. Most of the tentatively identified chlorogenic acids in our study were previously detected in *R. hirta* leaves by Jaiswal et al., 2011 [43].

Glycosides of *p*-coumaric acid and dihydrocoumaric acid (**10**, **13**, **14**, **18**, **22**, **25**), as well as conjugates of *p*-coumaric acid, ferulic acid and benzoic acid with glycerol and hexuronic acid (**39**, **46**, **56**), were detected [41] (clusters C-3 and C-4, Figure 4A). Simple hydroxycinnamic acids such as caffeic (**21**) and *p*-coumaric (**37**) were also identified.

*Rudbeckia* spp. represent a rich source of flavonoids. Methylated flavonols, i.e., quercetagetin 6-methylether (patuletin, C_16_H_12_O_8_), quercetagetin 3,6,7-trimethyl ether (chrysosplenol-D, C_18_H_16_O_8_), 6,7-dimethoxy-3,5,4′-trihydroxyflavone (eupalitin, C_17_H_14_O_7_), 6,7-dimethoxy-3,3′,4′,5-tetrahydroxyflavone (eupatolitin, C_17_H_14_O_8_), together with their *O*-glycosides or quercetin (C_15_H_10_O_7_) and quercetagetin (C_15_H_10_O_8_) 7-*O*-glucopyranosides were isolated or identified previously using the authentic reference standards in the leaves and flowers of *Rudbeckia hirta* [32,36,37,57]. 8-Hydroxyquercetin (gossypetin, C_15_H_10_O_8_) and kaempferol (C_15_H_10_O_6_) 7-*O*-glycosides, together with 3-*O*-glycosides of quercetin and kaempferol were also isolated from the flowers of *R. hirta* and aerial parts of *R. fulgida* and *R. laciniata* [33,37,61]. In our study, 39 flavonoid-*O*-glycosides and 5 flavonol aglycones were tentatively identified (over 23.2% of the total CAD peak area, second only to sesquiterpenoids) based on their MS/MS fragmentation patterns obtained in both ionization modes, the diagnostic retro Diels–Alder ions observed in the negative ionization mode, UV absorption maxima, and the published literature [41,62,63]. Among the flavonoids that were previously described in the *Rudbeckia* L. genus, numerous methylated flavonol glycosides were identified in our plant material (see cluster C-1, Figure 4A), such as eupalitin glycosides, which released the protonated aglycon fragment at *m*/*z* 331 and deprotonated aglycon radical at *m*/*z* 328 indicating 3-*O*-glycosides (**117**, **127**, **138, 170**) with a neutral loss of 162, 248, 146, 188 Da. Moreover, eight eupatolitin 3-*O*-glycosides (**100**, **101**, **121**, **124**, **128**, **142**, **152**, **153**) were determined regarding the observed deprotonated aglycon radical at *m*/*z* 344 and its protonated counterpart at *m*/*z* 347, sugar units were represented by deoxyhexose (−146 Da), hexose (−162 Da) or combination of them (−308 Da) or acetylated deoxyhexose (−188 Da), and acetylated hexose (−204 Da). Patuletin (6-methoxyquercetin) 7-*O*-hexoside (**75**) yielded a deprotonated aglycon ion at *m*/*z* 331 and its intensive fragment at *m*/*z* 315 and the neutral loss of 162 Da. 3-*O*-Dihexoside (**35**), -hexoside (**70**), -deoxyhexoside (**84**), -acetyldeoxyhexoside (**105**) of patuletin were also detected. Methylated flavanol aglycones, i.e., eupatolitin (**145**), chrysosplenol D (**175**), hexamethylquercetagetin (194), tangeritin (**208**) were established. Quercetin (**102**), its glycosides (**69**) and conjugate with *p*-coumaric acid (**104**) were also detected. Jaceidin (quercetagetin 3,3′,6-trimethyl ether) conjugates with hexose (**122**, **125**), deoxyhexose (**141**), hydroxymethylglutaryl-hexose (**133**) and malonyl-hexose (**134**), as well as flavone glycosides, i.e., eryodictyol-7-*O*-hexosides (**41**, **48**, **62**, **91**) and luteolin-7-*O*-malonyl hexoside (**50**, **54**) were identified for the first time in *Rudbekia hirta*. Biflavonoids with C-O linkage between luteolin, eriodyctiol and naringenin gave in the positive ionisation mode precursor ions at *m*/*z* 1317 (C₆₃H₆₄O₃₁, **58**, **59**, **63**, **68**) and *m*/*z* 883 (C_42_H_42_O_21_, **42**, **64**), *m*/*z* 885 (C_42_H_44_O_21_, **65**, **82**), and *m*/*z* 899 (C_42_H_42_O_22_, **83**), and formed a subfamily in the t-SNE output (Figure 2B).

### 2.2. Antioxidant Assays

The data obtained after performing the antioxidant assays revealed that the *Rudbeckia hirta* methanolic extract presented similar iron chelation capacity to that of quercetin, which was used as positive control, with EC_50_ values of 0.42 ± 0.00 and 0.42 ± 0.01 mg/mL final solution, respectively (Table 1).

On the other hand, the LOX inhibition activity showed promising results for the tested sample, with an EC_50_ of 48.18 ± 0.17 μg/mL final solution, but with a rather lower potency than that of quercetin. This could be attributed to various compounds present in the extract that either have no activity on the enzyme or present reversed action (Table 2).

### 2.3. Antimicrobial Testing

Regarding the activity shown on Gram-positive bacteria, the extract obtained from *Rudbeckia hirta* flowers (Rh-MeOH) proved to possess a good capacity for inhibiting bacterial growth (16.0 mm) (Table 3). Against Gram-negative bacteria, the extract showed a moderate action of growth-inhibiting (10.0 mm). Interestingly, the same extract presented a good antifungal action, with a notable activity on *Candida parapsilosis* (17.0 mm), similar to that observed for the used standards (20.0 mm for nystatin and 21.0 mm for fluconazole, respectively).

Regarding the determination of MIC and MBC values, it is generally observed that the MBC values are 2–4 times higher than those for MIC but much higher than those observed for ciprofloxacin, thus indicating a relatively low potency when compared to the referenced antibiotic (Table 4).

### 2.4. Cytotoxicity Evaluation

**Figure 5:**
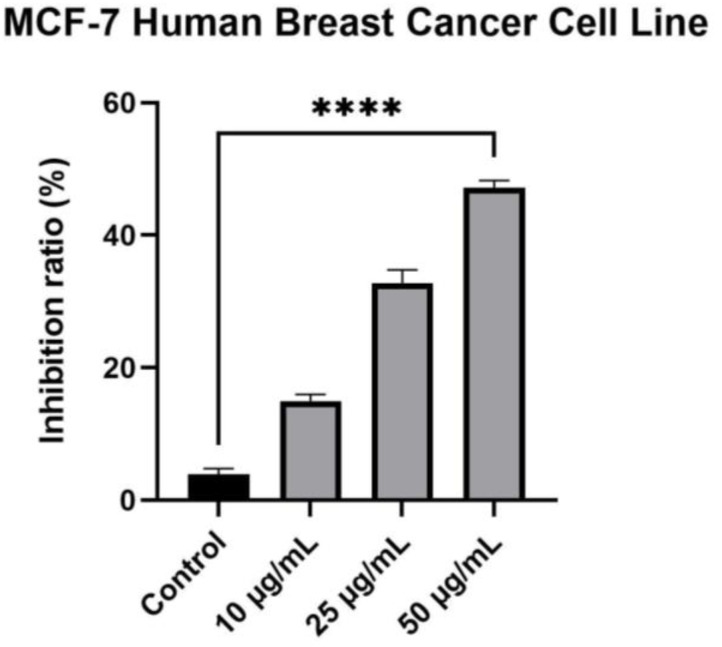
MCF-7 human breast cancer cells inhibition after exposure to different concentrations of *Rudbeckia hirta* extract (10, 25 and 50 µg/mL, respectively). The data is represented as mean ± SD. Comparison among groups was made using one-way ANOVA and Dunnett’s multiple comparison test (**** *p* < 0.0001 vs. control).

The extracts elicited a different activity among the tested breast cancer cell lines (estrogen receptor (ER)-positive MCF-7 and ER-negative MDA-MB-231 cells). Results have shown that the MCF-7 human breast cancer cell line is more sensitive, and this property is directly proportional to the concentration. For this cell line, at the tested concentrations, namely 10 μg/mL, 25 μg/mL and 50 μg/mL, the methanolic extract obtained from the flowers of *Rudbeckia hirta* presented promising activity, the inhibition ratio being directly proportional with the concentration, leading to a percentage of inhibition of 14.98 ± 1.00%, 32.71 ± 2.06% and 47.17 ± 1.13%, respectively (Figure 5).

**Figure 6:**
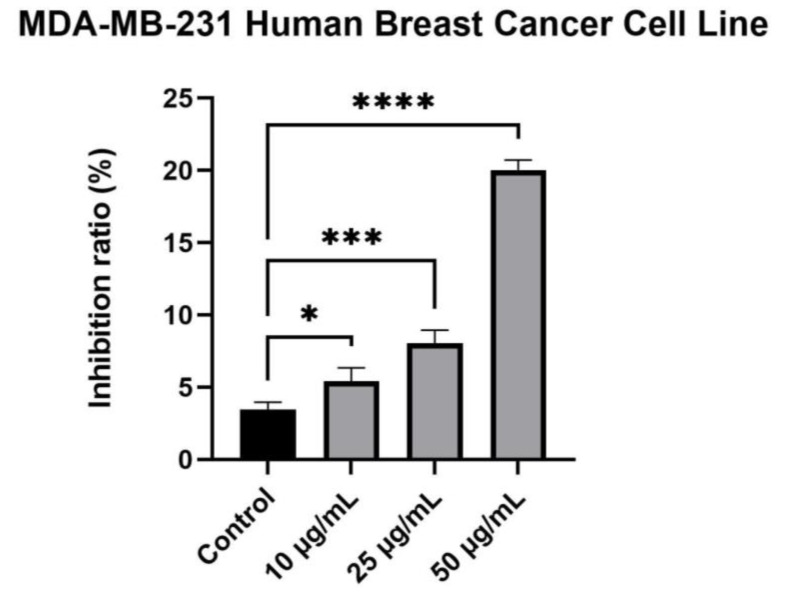
MDA-MB-231 human breast cancer cells inhibition after exposure to different concentrations of *Rudbeckia hirta* extract (10, 25 and 50 µg/mL, respectively). The data is represented as mean ± SD. Comparison among groups was performed using one-way ANOVA and Dunnett’s multiple comparison test (* *p* ≤ 0.1; *** *p* ≤ 0.001; **** *p* ≤ 0.0001 vs. control).

As previously mentioned, in the case of MDA-MB-231, lower percentages that correspond to inhibition were recorded. At the highest tested concentration, the inhibition ratio did not exceed 30% (Figure 6).

**Figure 7:**
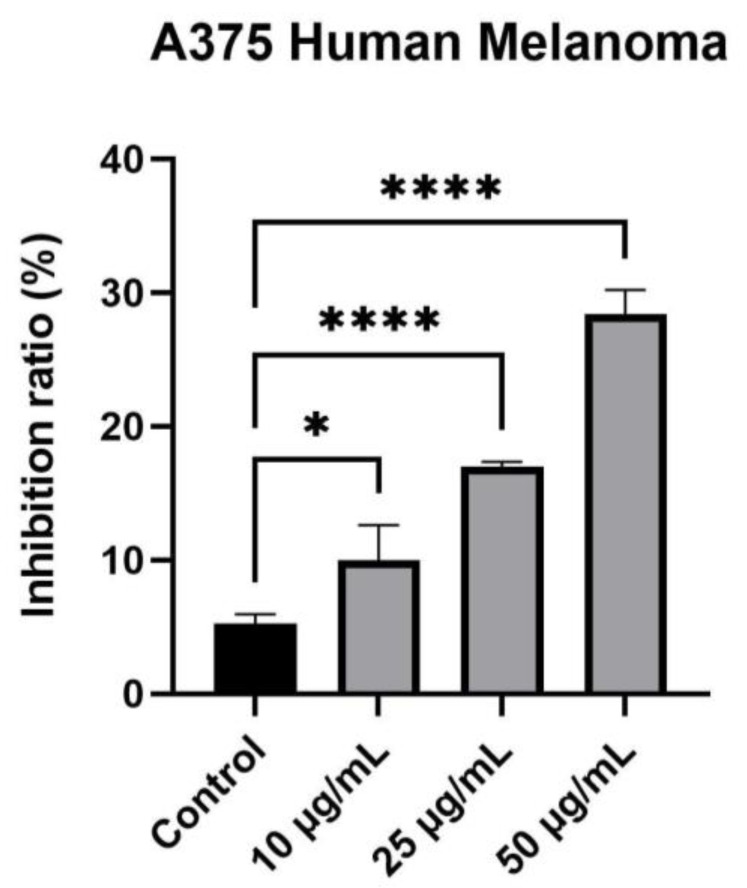
A375 human melanoma cell inhibition after exposure to different concentrations of *Rudbeckia hirta* extract (10, 25 and 50 µg/mL, respectively). The data is represented as mean ± SD. One-way ANOVA and Dunnett’s multiple comparison test were used to determine the significant differences between the control and treated groups (* *p* ≤ 0.1; **** *p* ≤ 0.0001 vs. control).

The behavior pattern for the A375 human melanoma cell line was similar to the one elicited by the MCF-7 human breast cancer cell line. However, in this case, only a weak antiproliferative effect of the studied extract was noticed at the tested concentrations (9.97 ± 2.63%, 16.99 ± 0.35% and 28.38 ± 1.85%, respectively) (Figure 7).

## 3. Discussion

The UHPLC-HR-MS phytochemical analysis and untargeted metabolite profiling performed on the *R. hirta* flower extract led to the identification of more than 250 compounds belonging to several classes of plant metabolites. Considering the relative content of compounds according to the CAD chromatogram, the most well-represented categories were: sesquiterpene lactones, flavonoid *O*-glycosides, amino acids and derivatives, quinic acid derivatives, coumaric acid derivatives, hydroxycinnamic acid glycosides, and fatty acids.

The human body constantly produces reactive oxygen species (ROS), such as superoxide radicals, hydroxyl radicals and hydrogen peroxide. These oxygen derivatives are normally neutralized by various physiological antioxidant mechanisms. However, an imbalance between ROS and antioxidants, either due to the overproduction of radicals or insufficient antioxidant defenses, can lead to DNA damage, lipid peroxidation, and protein carbonylation, all of which may contribute to the onset of certain diseases. Substances that have the potential to chelate Fe^2+^ ions act as antioxidants by reducing the availability of ferrous ions and, implicitly, of •OH ions generated during Fenton reactions [64,65]. This is one of the mechanisms by which compounds with functional groups such as hydroxyl, carbonyl and amino act as antioxidants. For example, flavonoids have a remarkable ability to chelate pro-oxidant metal ions such as Cu^2+^ or Fe^2+^, which contributes to their antioxidant activity [66]. Many members of this class can form stable complexes with such metals through the many OH groups they possess but also through the carbonyl group when present. Quercetin, for example, has the ability to form such stable complexes due to three potential metal ion binding sites: α-hydroxycarbonyl, β-hydroxycarbonyl and catechol groups [67]. The results obtained indicate a promising activity for the tested *R. hirta* extract, similar to that of quercetin. These results can be explained by a higher concentration of polyphenolic substances in the total extract, which increases the amount of free hydroxyl groups that can form complexes with ferrous ions.

In addition to their role in the development of inflammatory processes, lipoxygenases also serve as mediators of bronchoconstriction and hypersensitive responses. Therefore, LOX inhibitors may be of interest for modulating these phenomena and for controlling inflammation, gastrointestinal disorders, adverse cardiovascular reactions, and even cancer [68,69]. Many LOX inhibitors are expected to be antioxidants and act by scavenging free radicals. The interaction of naturally occurring polyphenolic chemicals with the enzyme is of particular interest, as it is thought to be a potential target for demonstrating the biological activity of such compounds. Consequently, LOX inhibitors have been investigated as potential therapeutics for the treatment of inflammatory and allergic diseases [68].

Compounds that prevent the reversible oxidation of Fe^2+^ to Fe^3+^ can partially or completely inhibit 15-LOX activity, preventing the enzyme from catalyzing the transformation of the substrate through redox reactions. When substances interact with the enzyme in this way, they exhibit reducing capacity, releasing electrons and protons [70,71]. Polyphenols, flavonoids and sesquiterpenes from the analyzed extract are the most likely compounds to exhibit inhibitory activity on the enzyme by the previously mentioned method, considering the chemical profile and the EC_50_ value obtained.

The few studies that have evaluated the antibacterial and antifungal activities of extracts from *R. hirta* have generally been conducted on other parts of the plant, such as leaves and roots [16,72]. In a study on the potential biological activities of the volatile oil obtained from leaves, Stewart et al. [72] tested its ability to inhibit the growth of several microorganisms. The data obtained in this research are in agreement with the values determined in the aforementioned study on *Staphylococcus aureus* and *Escherichia coli*, which illustrates the importance of continuing the study on the inflorescences of this species. However, in the present case, the antimicrobial activity can be attributed to both terpenoids and polyphenolic compounds present in the extract, and not to polyacetylenic compounds, which are considered responsible for the activity observed in previous studies. 

The antimicrobial capacity of sesquiterpene lactones may be related to the presence of a double α,β-unsaturated carbonyl group and an α-methylene-γ-lactone functional group. These compounds are thought to interact with membrane phospholipids and also alter protein synthesis. Gram-positive bacteria appear to be more susceptible to the effects of sesquiterpene lactones than Gram-negative bacteria, suggesting that the observed antimicrobial activity may be correlated with the presence of such compounds [73].

While the MDA-MB-231 cell line provides a model for more aggressive, hormone-independent breast cancer, the MCF-7 cell line has functional estrogen and EGF receptors, is dependent on estrogen and EGF for proliferation, and is noninvasive [74]. In addition, the MCF-7 cell line represents a model of early-stage disease, whereas the MDA-MB-231 cell line is often used as a model of advanced breast cancer [75]. The results obtained show a higher degree of inhibition on the MCF-7 cell line, which could indicate a promising anticancer activity of the extract at higher concentrations, considering that the percentage of inhibition was dose-dependent. To the best of our knowledge, this is the first report on the in vitro anticancer activity of a *R. hirta* flower extract. Only one previous study on this species reports the investigation on P388 cancer cells to ensure that compounds isolated from an extract are further used at subcytotoxic doses for in vitro immunomodulatory activity testing [37]. 

Sesquiterpene lactones are considered to be the main compounds responsible for the observed antitumor activity. This activity could be explained by three main characteristics: structural flexibility, high lipophilicity, and alkylating capacity. Moreover, the α-methylene-γ-lactone moiety seems to be essential for such biological effects. Various processes are disturbed when this type of structure reacts with nucleophilic groups, such as sulfhydryl groups present in enzymes and proteins. Therefore, sesquiterpene lactones have the ability to alter the redox state, selectively causing oxidative stress and death in cancer cells [76]. In addition, rudbeckolide (**168**), one of the major sesquiterpenoids identified in the studied methanolic extract, showed strong inhibitory activity in vitro against the pro-inflammatory enzyme 5-LOX [17]. The literature suggests that pseudoguaianolide-type lactones showed potent cytotoxic activity against breast cancer cells in vitro [77,78,79,80,81]. Qin et al., 2013 [77] described selective cytotoxicity towards the MCF-7 and MDA-MB-231 cell lines by monoacetylated pseudoguaianolides (bigelovin and 2α-acetoxy-4β-hydroxy-1αH,10αH-pseudoguai-11(13)-en-12,8β-olide) isolated from *Inula lineariifolia* Turcz (Asteraceae). Another monoacetylated lactone (aucherinolide) isolated from *Inula aucheriana* DC demonstrated high cytotoxicity against MCF-7 [78]. Non-acetylated form of pseudoguaianolides (hymenin, ambrosin, damsin) isolated from *Ambrosia maritima* L. (Asteraceae) showed moderate toxicity to MCF-7 [80]. The series of twenty-one 10α-methylpseudoguaianolides (helenanolide-type) and their esters isolated from *Arnica* species (Asteraceae) were tested in vitro against the cloned Ehrlich ascites tumor cell line, EN2 [79]. As a result, the esterified forms with short acyl chains (acetyl and isobutyryl) were more toxic than the helenanolides themselves. However, the tigloyl and isovaleryl esters showed reduced toxicity compared to the unesterified form.

The potent cytotoxicity of the studied methanolic extract towards the MCF-7 cell line could be explained by the presence of mono-, di-, tri-esterified sesquiterpene lactones with short or large acyl groups. Another group of the identified metabolites that may be responsible for the cytotoxicity of the *R. hirta* flower extract are methylated quercetagetin derivatives (eupatolitin, patuletin, etc.) [82,83,84].

## 4. Materials and Methods

### 4.1. Chemicals and Reagents

Formic acid was purchased from Sigma Aldrich (Steinheim, Germany), while acetonitrile (LC-MS grade) and methanol (HPLC grade) were acquired from Merck (Darmstadt, Germany). A Milli-Q Simplicity 185 water filtration system (Millipore, Milford, MA, United States) generated ultrapure water. Regarding the antioxidant activity evaluation, lipoxidase from soybean (type I-B), linoleic acid, quercetin, and dimethyl sulfoxide (DMSO) were purchased from Sigma Aldrich (Steinheim, Germany), and acetate buffer 0.1 M (pH 5.25) was prepared by combining sodium acetate with acetic acid (Sigma Aldrich) until the required pH was reached. Similar outcomes were achieved by combining boric acid (Sigma-Aldrich) and NaOH (1 N), thus obtaining borate buffer (pH 9). In addition, the ferrous sulfate solution in 0.2 M hydrochloric acid and the 5 mM ferrozine solution were prepared using chemicals purchased from Sigma Aldrich (Steinheim, Germany). 

Considering the cytotoxicity assays, Dulbecco’s Modified Eagle’s Medium, as well as the penicillin/streptomycin mixture, were acquired from Sigma Aldrich, while Eagle’s Minimum Essential Medium was purchased from ATCC. Moreover, the fetal bovine serum was acquired from ThermoFisher Scientific, Boston, MA, USA.

### 4.2. Plant Material and Extract Preparation

**Figure 8:**
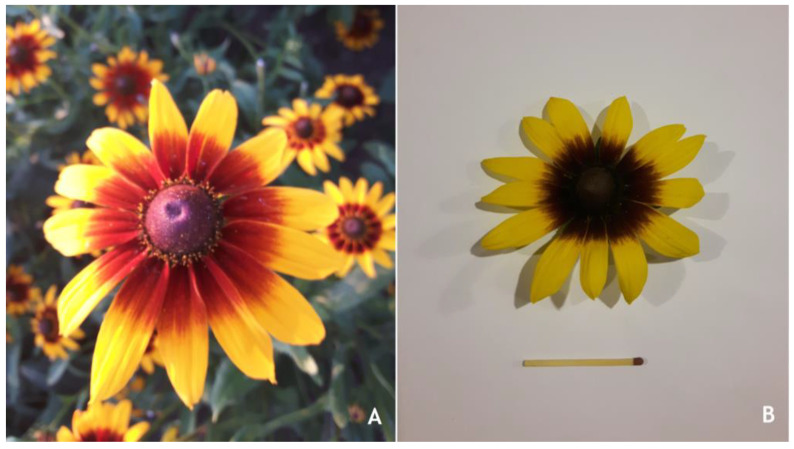
The cultivated plant species (**A**) and a freshly harvested flower (**B**).

The plant material was represented by *Rudbeckia hirta* (“Glor. Daesi” cultivar) flowers collected from plants that were cultivated in environmentally friendly conditions in northeastern Romania (Figure 8). 

The cultivated area measured approximately 50 m^2^. The soil in the area is considered to be clay loam, weakly carbonated, with a good humus content [85]. A whole foliar herbicide was applied to prepare the land for agriculture. The soil was cleared of weeds and foreign matter after approximately 10 days, during which time the existing vegetation had dried. The earth was then mechanically dug, after which followed soil loosening and raking. In order to facilitate numerous technical interventions (e.g., irrigation, weeding, harvesting), a unique rectangular plot was considered.

Seeds with a fast germination rate were used. After the seeds germinated and seedlings were obtained, they were planted in rows, with minimum distances between plants of 40 cm. The early half of May marked the planting process. Daily irrigation of the seedlings was provided, but no disease preventive or control treatments were applied. Moreover, cultivation was carried out in ecological conditions without the use of fertilizers. Hand-weeding was carried out periodically. The plant material, consisting of the inflorescences of the studied species, was harvested over the course of two weeks between July and August. Harvesting took place in dry conditions between 8:00 and 11:00 a.m., during the maximum development of the inflorescences.

The collected flowers were dried naturally in open spaces with low humidity, protected from sunlight [86]. The plant material was dried on a wooden bed covered with white paper. The drying process resulted in obtaining a constant mass of plant material, which was subsequently stored in paper bags. The plant material was later deposited in the Pharmacognosy department of “Grigore T. Popa” University of Medicine and Pharmacy Iași. 

For the obtaining of extracts, the flowers were ground using a commercial electric blender. The obtained finely ground material was weighed and mixed with methanol in a 1:20 ratio [87,88]. Extraction was carried out at room temperature for 3 h, using a magnetic stirrer (DLAB MS-M-S10, Beijing, China) [89]. Afterward, the extract was filtered using filter paper, and the methanol was evaporated using a Büchi R-210 rotavapor system (80 mbar, 40 °C). The obtained dry extract was stored at 4 °C till various tests were conducted.

### 4.3. LC-MS and Molecular Networking

HR-ESI-MS analysis of the crude methanolic extract of *Rudbeckia hirta* flowers was performed in positive and negative ionization modes using a liquid chromatograph Thermo Scientific Ultimate 3000 RS (Thermo Fischer Scientific, Waltham, MA, USA) equipped with a Diode Array Detector (DAD), a Charged Aerosol Detector (CAD), and coupled with a quadrupole time-of-flight (Q-TOF) high-resolution Bruker Impact II HD mass spectrometer (Bruker, Billerica, MA, USA). 

The chromatographic condition, stationary and mobile phases, MS fragmentation settings and data acquisition were obtained using the conditions described in our previous works [89,90]. 

Annotation of metabolite structures was supported by MS-DIAL (Version 4.9.221218)/MS-FINDER (Version 3.52) [19,20] and SIRIUS (Version 5.7.2) [21] in negative and positive ionization modes. The MS-DIAL parameters common to both modes were: MS1 tolerance of 0.01 Da; MS2 tolerance of 0.05 Da; minimum peak height 1000 (amplitude). The MS/MS public databases used for compound identification were MSMS_Public_EXP_Pos_VS17 and MSMS_Public_EXP_NEG_VS17. MS/MS spectra of compounds for which hits could not be found in the databases were subjected to in silico fragmentation analysis in MS-FINDER and SIRIUS. Finally, the pre-clustered spectral data files (in .mgf format) and their corresponding .csv metadata file (containing retention times, areas and chemical formulas) were exported from MS-DIAL using the dedicated “GNPS export” built-in option and processed with the MetGem software (Version 1.3.6) [22] to give networks containing nodes distributed in clusters. Networks were generated using the following parameters: *m*/*z* tolerance set to 0.01; minimum matched peaks set to 3 (negative ion mode) or 6 (positive ion mode); topK set to 10; minimal cosine score value of 0.7; max connected component size of 1000. T-SNE visualization was generated using the following parameters: at least 1 cosine score above 0.70; number of iterations set to 5000; perplexity 6 (negative ion mode) or 16 (positive ion mode); learning rate set to 200; early exaggeration set to 12; using Barnes-hut approximation with an angle set to 0.5.

### 4.4. Antioxidant Activity Assays

#### 4.4.1. Lipoxygenase Inhibition

The inhibition of the enzyme was evaluated using a modified Malterud method [70,91]. 50 μL *Glycine max* lipoxidase in borate buffer was treated with 50 μL of a sample/control solution of varying concentrations in DMSO and allowed to stand for 10 min. After adding 2 mL of 0.16 mM linoleic acid borate buffer, the absorbance was measured for 90 s at 234 nm. The evaluation was carried out at room temperature. Using the following formula, the inhibition of lipoxygenase was determined: Inhibition (%) = (*A_EFI_* − *A_ECI_*) × 100/*A_EFI_*, where *A_EFI_* is the difference between the absorbances of the enzyme solution without inhibitor at 90 and 30 s, respectively, whereas *A_ECI_* represents the difference between the enzyme-inhibitor solution absorbances at 90 and 30 s, respectively. Each experiment was conducted in triplicate. The positive control employed was quercetin, and IC_50_ values were expressed as µg/mL final solution. 

#### 4.4.2. Iron Chelation Capacity

The metal-chelating assay was evaluated according to the previously modified Venditti method [92]. The reaction between ferrous ions and ferrozine produces a pink complex with maximum absorbance at 562 nm. Hence, the presence of a chelating agent in the reaction media will decrease the recorded absorbance. Moreover, 0.2 mL sample solution, 0.74 mL acetate buffer 0.1 M and 0.02 mL ferrous sulfate solution (2 mM) in 0.2 M hydrochloric acid were mixed. After adding 0.04 mL of a 5 mM ferrozine solution, the absorbance of the solution was measured after 10 min against a blank solution similarly prepared. The metal chelating capacity was established using the following formula: Iron chelation capacity (%) = 100 × (*Ac* − *As*)/(*Ac*), where *Ac* is the absorbance of the control, whereas *As* is the absorbance of the sample. Experiments were conducted in triplicate. Quercetin served as a positive control, and the EC_50_ values were expressed as mg sample/mL final solution. 

### 4.5. Antimicrobial Testing

#### 4.5.1. Antimicrobial Susceptibility Testing

As part of the biological evaluation of the methanolic extract, antimicrobial susceptibility testing was carried out. Using Mueller–Hinton broth (Oxoid) for bacteria and Mueller–Hinton broth (HiMedia) for fungi, the antimicrobial activity was determined following a disk diffusion method previously described [93]. *Staphylococcus aureus* ATCC 25923, *Escherichia coli* ATCC 25922, *Pseudomonas aeruginosa* ATCC 27853, *Candida albicans* ATCC 90028 and *Candida parapsilosis* ATCC 22019 were the tested microorganisms that were available from the Culture Collection of the Microbiology Department, “Grigore T. Popa” University of Medicine and Pharmacy, Iași, Romania. 

Commercial discs containing ciprofloxacin (5 µg/disc), fluconazole (25 µg/disc) and nystatin (100 µg/disc) were used as standards. The plates were incubated for 24 h at 37 °C for bacteria and for 48 h at 24 °C for fungi. Following incubation, the inhibition zone diameters corresponding to the plant extract and the standards were measured in mm, including the size of the disc. All assays were performed in triplicate, and results are expressed as the arithmetic mean of three assays ± standard deviation.

#### 4.5.2. Determination of the Minimum Inhibitory Concentration (MIC) and the Minimum Bactericidal Concentration (MBC)

The determination of MIC and MBC values was done considering one Gram-positive and one Gram-negative bacterial strains (*S. aureus* ATCC 25923 and *E. coli* ATCC 25922, respectively), for which promising results were obtained. Dilutions of each extract in Mueller Hinton broth (Oxoid) were inoculated with equal volumes of bacterial or fungal suspension (10^6^ CFU/mL). MIC represents the lowest concentration of plant extract at which a complete inhibition of the visible growth of the microorganism was observed after incubation at 37 °C for 24 h. The MBC values were determined by transferring 0.1 mL of extract showing complete inhibition of visible growth on the surface of an agar plate. Subcultures were incubated at 37 °C for 24 h. MBC is the lowest extract concentration required to kill more than 99.9% of the tested microorganisms. The MIC and MBC of ciprofloxacin against the two bacterial strains were also evaluated.

### 4.6. Cytotoxicity Testing

MCF-7—human breast adenocarcinoma cell line (ATCC^®^ HTB-22TM), MDA-MB-231—human breast adenocarcinoma cell line (ATCC^®^ HTB-26TM) and A375—human melanoma cell line (ATCC^®^ CRL-1619TM) were acquired from the American Type Culture Collection (ATCC). MCF-7 cells were cultured in Eagle’s Minimum Essential Medium; MDA-MB-231 and A375 cells were cultured in high glucose Dulbecco’s Modified Eagle’s Medium. Each cell line was supplemented with 10% fetal bovine serum and 1% penicillin/streptomycin mixture (10,000 IU/mL). Standard conditions were used for cell culture—37 °C and humidified atmosphere containing 5% CO_2_ in a Steri-Cycle i160 incubator (ThermoFisher Scientific, Boston, MA, USA).

The assessment of the antiproliferative activity was conducted as previously described [94,95]. Using 96-well plates, the cells were plated at a density of 5000 cells per well and exposed to the tested extract at concentrations of 10 μg/mL, 25 μg/mL and 50 μg/mL, respectively, for 72 h. After the incubation period, 5 mg/mL MTT solution was added and incubated for another 4 h. Using a microplate reader, the absorbance of the precipitated formazan crystals dissolved in DMSO was measured at 545 nm. Cells cultured in wells with medium and DMSO served as control. The average of three different experiments is shown as the final result.

## 5. Conclusions

To the best of our knowledge, this is the first comprehensive study that includes data on the in vitro antioxidant, antimicrobial and anticancer activities of black-eyed Susan cultivated in Romania. The untargeted metabolite profiling and UHPLC-HR-MS phytochemical analysis of the extract obtained from *R. hirta* flowers revealed the presence of over 250 compounds pertaining to various groups of plant metabolites, such as sesquiterpene lactones, flavonoid *O*-glycosides, polyphenolic acids, amino acids and fatty acids. The investigated extract presented inhibitory activity against *Staphylococcus aureus* and promising antifungal and antioxidant activities. Moreover, the assessment of the cytotoxic activity indicated that the extract may possess anticancer effects by preventing cell growth under experimental conditions, especially on the MCF-7 cell line.

## Figures and Tables

**Table 1:** Chelation activity (%) and EC_50_ values of the tested extract (Rh-MeOH) and of the positive control.

Sample/ Control	Concentration (mg/mL)							EC_50_ (mg/mL Final Solution)
	0.15625	0.3125	0.625	1.25	2.5	5	10	
Rh-MeOH	6.23 ± 0.05	11.65 ± 0.03	18.02 ± 0.09	29.08 ± 0.01	56.31 ± 0.03	69.70 ± 0.06	94.95 ± 0.08	0.42 ± 0.00
Quercetin	3.92 ± 0.07	6.63 ± 0.28	16.03 ± 0.38	31.60 ± 1.30	56.48 ± 0.83	75.59 ± 0.79	90.35 ± 0.92	0.42 ± 0.01

**Table 2:** LOX inhibition (%) and EC_50_ values of the tested extract (Rh-MeOH) and of the used control.

Sample/ Control	Concentration (mg/mL)							EC_50_ (μg/mL Final Solution)
	0.15625	0.3125	0.625	1.25	2.5	5	10	
Rh-MeOH	13.67 ± 0.60	16.80 ± 0.36	19.36 ± 0.29	26.12 ± 0.65	36.73 ± 0.40	100 ± 0.00	100 ± 0.00	48.18 ± 0.17
Quercetin	25.33 ± 0.42	38.40 ± 0.36	53.98 ± 0.46	63.08 ± 0.86	77.45 ± 1.12	100 ± 0.00	100 ± 0.00	17.45 ± 0.33

**Table 3:** Antimicrobial activity of the extract and positive controls.

Sample/ Control	Inhibition Zone Diameter (mm)				
	*S. aureus* ATCC 25923	*E. coli* ATCC 25922	*P. aeruginosa* ATCC 27853	*C. albicans* ATCC 90028	*C. parapsilosis* ATCC 22019
Rh-MeOH	16.0 ± 0.00	10.0 ± 0.00	10.0 ± 0.00	15.0 ± 0.00	17.0 ± 0.00
DMSO	0	0	0	0	0
Ciprofloxacin	28.7 ± 0.06	36.5 ± 0.50	31.5 ± 0.50	*NT	*NT
Fluconazole	*NT	*NT	*NT	30.5 ± 0.50	21.0 ± 0.00
Nystatin	*NT	*NT	*NT	23.5 ± 0.50	20.0 ± 0.00

*NT—not tested.

**Table 4:** MIC and MBC values of the tested extract against the tested bacterial strains.

Sample/Control	*Staphylococcus aureus* ATCC 25923		*Escherichia coli* ATCC 25922	
	MIC (mg/mL)	MBC (mg/mL)	MIC (mg/mL)	MBC (mg/mL)
Rh-MeOH	0.25	0.5	0.25	>0.5
Ciprofloxacin	1 *	1 *	1 *	2 *

* µg/mL.

## Data Availability

Not applicable.

## Supplementary Materials

The following supporting information can be downloaded at: https://www.mdpi.com/article/10.3390/plants12152871/s1, Table S1: Tentative identification of compounds found in *R. hirta*, Table S2: Relative %CAD area of groups of compounds found in *R. hirta.*

## Abbreviations

ATCC	American Type Culture Collection
CAD	Charged aerosol detector
DAD	Diode array detector
DMSO	Dimethyl sulfoxide
EC_50_	Efficient concentration 50
EGF	Epidermal growth factor
ER	Estrogen receptor
ESI	Electrospray Ionisation
GNPS	Global Natural Products Social
HRMS	high-resolution mass spectrometry
IC_50_	Inhibition concentration 50
LC-MS	Liquid chromatography–mass spectrometry
LOX	Lipoxygenase
MBC	Minimum bactericidal concentration
MIC	Minimum inhibitory concentration
MS	Mass spectrometry
MTT	3-(4,5-dimethylthiazol-2-yl)-2,5-diphenyltetrazolium bromide
PDA	Photodiode Array Detector
Q-TOF	Quadrupole Time-of-Flight
t-SNE	T-distributed stochastic neighbor embedding
UHPLC-HR-MS	Ultra-High-Performance Liquid Chromatography–High-Resolution Mass Spectrometry
