# Supplementary material for: Preliminary Phytochemical and Biological Evaluation of Rudbeckia hirta Flowers
Source: Plants (Basel). 2023 Aug 4;12(15):2871. doi: 10.3390/plants12152871 (PMC10420942; doi:10.3390/plants12152871)
Supplement: Supplementary file 1 [file plants-12-02871-s001.zip › plants-2515187-supplementary.pdf]

plants

MDPI

Supplementary material

Preliminary Phytochemical and Biological Evaluation of *Rudbeckia hirta* Flowers

Ana Flavia Burlec [1,†], Łukasz Pecio [2,3,†], Cornelia Mircea [1,*], Cristina Tuchiluș [4], Andreia Corciovă [1,*], Corina Danciu [5], Oana Cioancă [1], Ioana Cezara Caba [1], Solomiia Pecio [2], Wiesław Oleszek [2] and Monica Hăncianu [1]

[1] Faculty of Pharmacy, “Grigore T. Popa” University of Medicine and Pharmacy, 16 University Street, 700115 Iasi, Romania; ana-flavia.l.burlec@umfiasi.ro (A.F.B.); oana.cioanca@umfiasi.ro (O.C.); ioana-cezara.caba@umfiasi.ro (I.C.C.); mhancianu@yahoo.com (M.H.)
[2] Department of Biochemistry and Crop Quality, Institute of Soil Science and Plant Cultivation—State Research Institute, Czartoryskich 8 Street, 24-100 Puławy, Poland; lpecio@iung.pulawy.pl (Ł.P.); skozachok@iung.pulawy.pl (S.P.); wieslaw.oleszek@iung.pulawy.pl (W.O.)
[3] Department of Chemistry of Natural Products, Medical University of Lublin, 1 Chodźki Street, 20-093 Lublin, Poland
[4] Faculty of Medicine, “Grigore T. Popa” University of Medicine and Pharmacy, 16 University Street, 700115 Iasi, Romania; cristina.tuchilus@umfiasi.ro
[5] Department of Pharmacognosy, University of Medicine and Pharmacy “Victor Babes”, Eftimie Murgu Square, No. 2, 300041 Timisoara, Romania; corina.danciu@umft.ro
* Correspondence: corneliamircea@yahoo.com (C.M.); acorciova@yahoo.com (A.C.)
† These authors contributed equally to this work.

Abstract: Black-eyed Susan (*Rudbeckia hirta* L.), a flowering plant with various traditional medicinal uses, has recently garnered interest for its therapeutic properties. However, little is known about the potential therapeutic activities of the plant species. The current study focused on conducting a comprehensive investigation into the chemical composition and bioactivity of black-eyed Susan cultivated in Romania. Untargeted metabolite profiling and UHPLC-HR-MS phytochemical analysis of the studied extract revealed the presence of more than 250 compounds pertaining to different classes, including sesquiterpene lactones, polyphenolic acids, flavonoids, amino acids, and fatty acids. The tested extract exhibited inhibitory activity against Gram-positive bacteria and showed promising antifungal activity. It also demonstrated potent antioxidant properties through iron chelation and 15-LOX inhibition capacities, as well as inhibition of cell growth, particularly on the MCF-7 cell line, suggesting potential anticancer effects. Therefore, current research provides valuable information on the antioxidant, antimicrobial, and antitumor potential of *Rudbeckia hirta* flowers. Implicitly, the discovery of such a wide range of biosubstances, together with the biological activity observed for the studied extract in these preliminary in vitro studies, paves the way for future investigation of the potential application of the plant in the pharmaceutical and nutraceutical sectors.

Keywords: black-eyed Susan; methanolic extract; UHPLC-HR-MS; sesquiterpenoids; phenolics; fatty acids; antioxidant activity; antimicrobial activity; MCF-7 cell line

Table S1. Tentative identification of compounds found in *R. hirta*.

No	*-MetGem Cluster*	*+MetGem Cluster*	*Rt (min)*	*Identification*	*Chemical ontology*	*Mol. formula*	*- ESI m/z*	*+ ESI m/z*	*-Δ* [a]	*+Δ*	*MS/MS ESI (-) m/z* [b]	*MS/MS ESI (+) m/z*	*UV/Vis (nm)*	*Ref.*	*Library Hit*
234		C2	23.29	Dehydrophytosphingosine isomer I	1,3-aminoalcohols	$C_{18}H_{37}NO_3$	n.d.	316.2849	n.d.	-0.89	n.d.	**316.2846**, 298.2745, 280.2641, 191.1803, 159.1193, 109.0640	n.d.		*Dehydrophytosphingosine*
237		C2	23.69	Dehydrophytosphingosine isomer II	1,3-aminoalcohols	$C_{18}H_{37}NO_3$	n.d.	316.2845	n.d.	0.38	n.d.	**316.2848**, 298.2742, 280.2635, 262.2546, 245.2285	n.d.		*Dehydrophytosphingosine*
246		C2	24.67	D-ribo-Phytosphingosine	1,3-aminoalcohols	$C_{18}H_{39}NO_3$	n.d.	318.2998	n.d.	1.48	n.d.	**318.2994**, 300.2896, 282.2782, 135.1171	n.d.		*D-ribo-Phytosphingosine*
240	Other		24.05	1-Octadecatrienoyl-sn-glyc-ero-3-phosphoethanolamine	1-acyl-sn-glycero-3-phosphoethanolamines	$C_{23}H_{41}NO_7P$	474.2625	n.d.	0.24	n.d.	474.2630, **400.2236**, 214.0494, 171.0067	n.d.	n.d.		*1-18:3-lysoPE (in silico)*
257	Other		25.44	1-Octadecadienoyl-sn-glycero-3-phosphoethanolamine	1-acyl-sn-glycero-3-phosphoethanolamines	$C_{23}H_{44}NO_7P$	476.278	n.d.	0.55	n.d.	476.2786, 402.2406, **171.0062**, 152.9932	n.d.	n.d.		*LysoPE(18:2(9Z,12Z)/0:0) (in silico)*
258	Other		25.92	1- Octadecadienoyl-sn-glyc-ero-3-phospho- inositol	1-acyl-sn-glycerol-3-phosphoinositols	$C_{27}H_{49}O_{12}P$	595.29	n.d.	-1.87	n.d.	**279.2328**, 241.0121, 152.9942	n.d.	n.d.		*1-linoleoyl-sn-glycero-3-phos-pho-D-myo-inositol (in silico)*
97		Other	9.27	Dehydrodiconiferyl alcohol 4-*O*-hexoside	2-arylbenzofuran flavo-noids	$C_{26}H_{32}O_{11}$	n.d.	503.1914 [M-H2O+H]+	n.d.	-0.44	n.d.	503.1952, 341.1388, 323.1284, 311.1271, 291.1010, 279.1023, 175.0753, 163.0757, **137.0598**	n.d.		*In silico*
180		Other	17.86	4'-Hydroxy-3'-phenylaceto-phenone	Alkyl-phenylketones	$C_{13}H_{16}O_2$	n.d.	205.1216	n.d.	3.46	**n.d.**	205.1218, 186.9562, **149.0594**, 121.0651	n.d.		*1-[4-hydroxy-3-(3-methylbut-2-enyl)phenyl]ethanone*
2	Other		0.75	*N*-(1-Deoxy-1-fructosyl)py-roglutamate	Amino acids	$C_{11}H_{17}NO_8$	290.088	n.d.	0.48	n.d.	200.056, 191.0560, 182.0445, **128.0340**	n.d.	n.d.		*N-Fructosyl pyroglutamate*

4		Other	0.77	Tyrosine	Amino acids	$C_9H_{11}NO_3$	n.d.	182.0808	n.d.	2.04	n.d.	182.0810, 165.0543, 147.0438, **136.0755**, 123.0437, 119.0489	n.d.	*L-TYROSINE*
5	Other	C4	0.87	*N*-(1-Deoxy-1-fructosyl) isoleucine	Amino acids	$C_{12}H_{23}NO_7$	292.1397	294.1542	1.62	1.8	130.0859	**276.1435**, 258.1331, 248.1484, 230.1379, 144.1014, 132.1012	n.d.	*N-Fructosyl isoleucine*
7		Other	0.97	Glutaryl-Leucine	Amino acids	$C_{11}H_{19}NO_5$	n.d.	246.1333	n.d.	1.22	n.d.	246.1315, 228.1240, 200.1285, **132.1013**	n.d.	*Glutaryllecucine (in silico)*
8		Other	1.21	Phenylalanine	Amino acids	$C_9H_{11}NO_2$	n.d.	166.0856	n.d.	4	n.d.	166.0857, **120.0804**	n.d.	*L-Phenylalanine*
9		C4	1.26	N-(1-Deoxy-1-fructosyl)phenylalanine	Amino acids	$C_{15}H_{21}NO_7$	326.1241	328.1384	1.3	2.07	206.0863, **164.0715**, 147.0446	**310.1283**, 292.1174, 264.1226, 178.0861, 166.0859, 132.0808	n.d.	*N-Fructosyl phenylalanine*
15	Other	Other	1.97	Tryptophan	Amino acids	$C_{11}H_{12}N_2O_2$	203.0827	205.0965	-0.48	3.2	**203.0825**, 186.0548, 142.0644, 116.0491	**188.0699**, 170.0593, 159.0909, 146.0595, 144.0803, 132.0804, 118.0644	285	*Tryptophan*
20		Other	2.86	Glutamylphenylalanine	Amino acids	$C_{14}H_{18}N_2O_5$	n.d.	295.1282	n.d.	2.2	n.d.	295.1290, 278.1012, 232.0967, **166.0854**, 120.0798	n.d.	*Glutamylphenylalanine (isomer of 1503)*
30	Other		3.78	*N*-Phenylacetylaspartic acid	Amino acids	$C_{12}H_{13}NO_5$	250.0718	n.d.	1.18	n.d.	132.0279	n.d.	n.d.	*Phenylacetylaspartic acid*
33		Other	3.98	3-(glutathion-S-yl)-hexan-1-ol	Amino acids	$C_{16}H_{29}N_3O_7S$	n.d.	408.1798	n.d.	1.84	n.d.	408.1808, 333.1462, 279.1354, **262.1093**, 245.0612, 162.0218	n.d.	*3-(glutathion-S-yl)-hexan-1-ol (in silico)*
60	Other	C11	5.9	*N*-malonyltryptophan	Amino acids	$C_{14}H_{14}N_2O_5$	245.0933 $[M\text{-}CO_2\text{-}H]^-$	n.d.	-0.55	n.d.	245.0925, **203.0821**, 201.1042	n.d.	n.d.	*Malonyltryptophan*

78	Other	Other	7.81	Amaronol B	Auronols	$C_{16}H_{14}O_8$	333.0608	335.0757	2.37	1.33	197.0449, **165.0180**, 137.0233, 121.0276	270.6704, 199.0606, 167.0319, **137.0230**, 109.0286	280		*in silico*
66		C2	6.57	Loliolide	Benzofurans	$C_{11}H_{16}O_3$	n.d.	197.1165	n.d.	3.68	n.d.	**197.1167**, 179.1058, 161.0951, 135.1164, 107.0847	n.d.		*Loliolide*
73		Other	7.3	5-hydroxyculmorin isomer I	Bicyclic monoterpenoids	$C_{15}H_{26}O_3$	n.d.	237.1844	n.d.	2.15	n.d.	237.1814, 219.1756, 201.1637, **151.1125**	n.d.		*5-hydroxyculmorin*
96		C2	9.25	5-hydroxyculmorin isomer II	Bicyclic monoterpenoids	$C_{15}H_{26}O_3$	n.d.	237.1847	n.d.	0.81	n.d.	237.1814, 219.1756, 201.1637, **151.1125**			*5-hydroxyculmorin*
113		C2	11.6	5-hydroxyculmorin isomer III	Bicyclic monoterpenoids	$C_{15}H_{26}O_3$	n.d.	237.1853	n.d.	-1.55	n.d.	**237.1858**, 219.1743, 201.1643, 191.1793, 145.1013	n.d.		*In silico*
42		C3	4.62	Hexosylphloretin-(I-4, O, II-2′)-luteolin-5-O-hexoside isomer I	Biflavonoids and polyflavonoids	$C_{42}H_{42}O_{21}$	n.d.	883.2269	n.d.	2.53	n.d.	**883.2264**, 721.1754, 559.1216, 433.0884, 423.0702, 285.0768, 273.0757	n.d.	[28]	*in silico*
58		C3	5.45	Bis(hexosylphloretin)-luteolin-5-O-hexoside isomer I	Biflavonoids and polyflavonoids	$C_{63}H_{64}O_{31}$	n.d.	1317.3441	n.d.	4.89	n.d.	**1317.3447**, 1155.2921, 10298.2627, 993.2402, 867.2114, 705.1611, 433.0926, 273.0734	n.d.		*in silico*
59		C3	5.79	Bis(hexosylphloretin)-luteolin-5-O-hexoside isomer II	Biflavonoids and polyflavonoids	$C_{63}H_{64}O_{31}$	n.d.	1317.3441	n.d.	4.89	n.d.	**1317.3466**, 1155.2921, 993.2347, 867.2113, 705.1506, 569.1072, 285.0736, 273.0754	n.d.		*in silico*
63		C3	6.1	Bis(hexosylphloretin)-luteolin-5-O-hexoside isomer III	Biflavonoids and polyflavonoids	$C_{63}H_{64}O_{31}$	n.d.	1317.3452	n.d.	4.13	n.d.	**1317.3441**, 1155.2919, 1029.2653, 993.2426,	n.d		*in silico*

												867.2131, 705.1582, 435.1330, 273.0769			
64		C3	6.27	Hexosylphloretin-(I-4, O, II-2′)-luteolin-5-O-hexoside isomer II	Biflavonoids and polyflavonoids	$C_{42}H_{42}O_{21}$	n.d.	883.2283	n.d.	0.95	n.d.	**883.2280**, 721.1747, 559.1240, 433.0920, 423.0729, 313.0705, 273.0745	n.d.	[28]	*in silico*
65		C10	6.42	Hexosylluteoliflavan-(4→8)-eriodictyol-hexopyranoside isomer I	Biflavonoids and polyflavonoids	$C_{42}H_{44}O_{21}$	n.d.	885.2415	n.d.	3.72	n.d.	721.1772, 561.1369, 435.1234, 409.0945, **273.0754**, 247.0595, 147.0445	n.d.	[29]	*In silico*
68		C3	6.77	Bis(hexosylphloretin)-luteolin-5-O-hexoside isomer IV	Biflavonoids and polyflavonoids	$C_{63}H_{64}O_{31}$	657.1641 $[M-2H]^{2-}$	1317.345	0.31	**4.13**	**548.0737**, 401.0298, 284.0331, 135.0439	**1317.3447**, 1155.2930, 1029.2604, 993.2412, 867.2115, 273.0728	n.d.		*In silico*
82		C10	8.14	Hexosylluteoliflavan-(4→8)-eriodictyol-hexoside isomer II	Biflavonoids and polyflavonoids	$C_{42}H_{44}O_{21}$	n.d.	885.2427	n.d.	2.36	n.d.	723.1882, 561.1383, 435.1279, 425.0802, 409.0955, 289.0703, **273.0754**, 247.0604, 163.0390	n.d.	[29]	*In silico*
83		C10	8.24	Hexosylnaringenin-(taxifolin-hexoside)	Biflavonoids and polyflavonoids	$C_{42}H_{42}O_{22}$	n.d.	899.2213	n.d.	3.06	n.d.	737.1705, 575.1182, 465.0992, 449.0848, 435.1283, 423.0728, 303.0503, **273.0750**, 247.0608, 147.0437	n.d.		*in silico*
143	Other	C5	14.14	*N*¹,*N*⁵,*N*¹⁰-Tri-*p*-coumaroyl spermidine	Coumaric acids and derivatives	$C_{34}H_{37}N_3O_6$	582.2609	584.2742	0.1	2.25	462.2062, 342.1463, **335.2231**, 316.1668, 299.1381, 119.0499	584.2737, **438.2375**, 420.2273, 275.1744, 204.1012, 147.0433	295		*Tricoumaroyl spermidine*

155		C5	15.31	Monocaffeoyl-tri-*p*-coumaroyl spermine	Coumaric acids and derivatives	$C_{46}H_{50}N_4O_9$	801.3494	803.363	1.37	2.56	545.2408, 502.2334, 459.2303, **399.2033**, 356.1970, 313.1923, 161.0215, 145.0275, 135.0430, 119.0501	803.3630, **657.3262**, 639.3156, 511.2903, 494.2620, 478.2678	295	*in silico*
156		C5	15.33	Tetra-*p*-coumaroyl spermine isomer I	Coumaric acids and derivatives	$C_{46}H_{50}N_4O_8$	n.d.	787.3682	n.d.	2.47	n.d.	787.3671, **641.3319**, 623.3193, 478.2654, 204.1021	n.d.	*N1,N5,N10,N14-Tetra-trans-p-coumaroylspermine*
161	C5		15.61	Tetra-*p*-coumaroyl spermine isomer II	Coumaric acids and derivatives	$C_{46}H_{50}N_4O_8$	785.3548	n.d.	1	n.d.	545.2410, **502.2347**, 459.2262, 399.2029, 356.1967, 313.1935, 213.8379, 119.0509	n.d.	n.d.	*N1,N5,N10,N14-Tetra-trans-p-coumaroylspermine*
165	C5	C5	15.99	Tetra-*p*-coumaroyl spermine isomer III	Coumaric acids and derivatives	$C_{46}H_{50}N_4O_8$	785.3557	787.3674	-0.14	3.49	545.2406, 502.2356, **399.2036**, 356.1985, 145.0289, 119.0486	787.3680, **641.3327**, 623.3214, 478.2706, 275.1746, 147.0430		*N1,N5,N10,N14-Tetra-trans-p-coumaroylspermine*
167	C5	C5	16.18	Tetra-*p*-coumaroyl spermine isomer IV	Coumaric acids and derivatives	$C_{46}H_{50}N_4O_8$	785.3548	787.3674	1	3.49	545.2410, 502.2341, 459.2288, **399.2043**, 353.1880, 145.0282, 119.0496	787.3675, **641.3314**, 623.3209, 478.2692, 275.1745, 204.1011	300	*N1,N5,N10,N14-Tetra-trans-p-coumaroylspermine*
53		C1	5.21	Acetyl rudbeckin A hexoside	Sesquiterpene lactones	$C_{23}H_{36}O_{11}$	533.2235 $[M+FA-H]^-$	506.2592 $[M+NH_4]^+$	0.95	0.79	n.d.	506.2552, **327.1796**, 309.1690, 267.1581, 249.1478, 237.1475, 231.1372, 219.1366, 203.1424	n.d.	
115	C8		11.64	4'-Desulfo-4-carboxyatractyloside isomer I	Diterpene glycosides	$C_{31}H_{46}O_{15}S$	689.2482	n.d.	0.38	n.d.	645.2615, **627.2476**, 543.1914, 525.1798, 463.2350, 343.0706, 301.1816, 241.0018	n.d.	n.d.	*in silico*

118	C8		11.82	4'-Desulfo-4-carboxyatractyloside isomer II	Diterpene glycosides	$C_{31}H_{46}O_{15}S$	689.2488	n.d.	-0.49	n.d.	645.2578, **627.2474**, 543.1899, 525.1807, 463.2349, 343.0713, 301.1807, 241.0021	n.d.	n.d.		*in silico*
129	Other	Other	12.64	3',4'-Didesulfo-4-carboxy-atractyloside	Diterpene glycosides	$C_{31}H_{46}O_{12}$	609.2905	628.3331	1.89	-0.57	481.2379, 463.2346, **301.1809**	365.1999, 347.1862, 329.1742, 283.1685, **247.1177**, 211.0967	n.d.		*in silico*
148	C8		14.66	Acetyl-4'-desulfo-4-carboxy-atractyloside	Diterpene glycosides	$C_{33}H_{48}O_{16}S$	731.2578	n.d.	1.68	n.d.	687.2698, **669.2587**, 585.2014, 567.1902, 385.0799, 301.1811, 283.0129, 222.9915, 176.9873	n.d.	n.d.		*in silico*
107	Other	Other	11.22	Pterodontoside E/F (pterodontriol B hexoside) isomer I	Eudesmane glycosides	$C_{21}H_{38}O_8$	463.2546 [M+FA-H]$^-$	441.2465 [M+Na]$^+$	0.65	-1.46	n.d.	**441.2464**, 263.1980, 203.0525	n.d.	[30]	*Ophiopogonoside A*
131	Other	C8	12.96	Pterodontoside E/F (pterodontriol B hexoside) isomer II	Eudesmane glycosides	$C_{21}H_{38}O_8$	463.2549 [M+FA-H]$^-$	419.2638	-0.07	0.35	n.d.	257.2116, 239.2003, **221.1893**, 203.1789, 143.1062, 135.1165	n.d.		
135		C8	13.2	Pterodontriol B malonyl-hexo-side	Eudesmane glycosides	$C_{24}H_{40}O_{11}$	459.2603 [M-CO$_2$-H]$^-$	487.2529 [M-H$_2$O+H]$^+$	-0.68	1.73	n.d.	**221.1895**, 203.1789, 143.1058, 135.1166	n.d.		
77		Other	7.77	Dihydroxy-dimethyldode-cenedioic acid	Fatty acids	$C_{14}H_{24}O_6$	n.d.	289.1645	n.d.	0.23	n.d.	253.1414, **235.1325**, 217.1218, 211.1328, 193.1216			
87	C2	Other	8.32	Azelaic acid	Fatty acids	$C_9H_{16}O_4$	187.0974	171.1011 [M-H$_2$O+H]+	0.44	2.5	**187.0972**, 169.086, 125.0965	**125.0959**	n.d.		*Azelaic acid*

126		C2	12.52	Tridecenynedioic acid isomer I	Fatty acids	$C_{13}H_{18}O_4$	n.d.	239.1277	n.d.	0.36	n.d.	239.1272, 203.1434, **179.1063**, 161.0960, 135.1164, 107.0854	n.d.	*In silico*
132		C2	12.98	Tridecenynedioic acid isomer II	Fatty acids	$C_{13}H_{18}O_4$	n.d.	239.1274	n.d.	2.04	n.d.	239.1278, 203.1783, **179.1062**, 161.0956, 135.1167, 107.0853	n.d.	*In silico*
136	Other		13.21	Hydroxy-hexadecanedioic acid	Fatty acids	$C_{16}H_{30}O_5$	301.2021	n.d.	-0.17	**n.d.**	**301.2021**, 265.1802, 201.1129, 183.1015, 155.1060	n.d.	n.d.	*in silico*
157	C6		15.45	Trihydroxy-octadecadienoic acid isomer I (9 (S*),12 (S*),13 (S*)-trihydroxyoctadeca-10(E),15(Z)-dienoic acid)	Fatty acids	$C_{18}H_{32}O_5$	327.2179	n.d.	-0.62	n.d.	327.2179, 293.2114, 229.1446, 221.1206, 211.1360, **201.1135**, 171.1021	n.d.	n.d.	*9S,10S,11R-trihydroxy-12Z,15Z-octadecadienoic acid (in silico)*
158	C6		15.46	Trihydroxy-octadecenoic acid isomer I	Fatty acids	$C_{18}H_{34}O_5$	329.2329	n.d.	1.36	n.d.	329.2336, 293.2114, **201.1129**	n.d.	n.d.	*12-Octadecenoic acid, 9,10,11-trihydroxy- (in silico)*
162	C2	C2	15.64	Trihydroxy-octadecadienoic acid isomer II (9 (S*),12 (S*),13 (S*)-trihydroxyoctadeca-10(E),15(Z)-dienoic acid)	Fatty acids	$C_{18}H_{32}O_5$	327.2176	346.2581 $[M+NH_4]^+$	0.3	2.13	327.2175, 291.1965, 229.1444, 221.1184, **211.1337**, 183.1386, 171.1022	293.2095, **275.1995**, 257.1896, 213.1480, 195.1372, 155.1060	n.d.	*(10E,15Z)-9,12,13-trihydroxyoctadeca-10,15-dienoic acid*
164	C2		15.97	Trihydroxy-octadecadienoic acid isomer III (9 (S*),12 (S*),13 (S*)-trihydroxyoctadeca-10(E),15(Z)-dienoic acid)	Fatty acids	$C_{18}H_{32}O_5$	327.2181	n.d.	-1.23	n.d.	327.2172, 291.1971, 229.1447, 221.1191, **211.1338**, 197.1208, 183.1355, 171.1017	n.d.	n.d.	*9,12,13-trihydoxy-10,15-octa-decadienoic acid (in silico)*
172	C2	C2	17.06	Trihydroxy-octadecenoic acid isomer II	Fatty acids	$C_{18}H_{34}O_5$	329.2334	313.2366 $[M-H_2O+H]^+$	-0.16	2.23	329.2333, 229.1444, **211.1340**, 183.1392, 171.1027	295.2261, **277.2159**, 259.2041, 195.1376, 165.1275	n.d.	*(Z)-5,8,11-trihydroxyoctadec-9-enoic acid*

173	C2	17.17	Trihydroxy-octadecenoic acid isomer III	Fatty acids	$C_{18}H_{34}O_5$	329.2333	n.d.	0.45	n.d.	*329.2340, 293.2122, 229.1447,* ***211.1340****, 171.1028, 139.1116*	n.d.	n.d.	*(Z)-5,8,11-trihydroxyoctadec-9-enoic acid*
174	C2	17.32	Trihydroxy-octadecenoic acid isomer IV	Fatty acids	$C_{18}H_{34}O_5$	329.234	n.d.	-1.67	n.d.	*329.2341, 293.2130, 229.1449,* ***211.1342****, 183.1397*	n.d.	n.d.	*(Z)-5,8,11-trihydroxyoctadec-9-enoic acid*
176	C2	17.5	Trihydroxy-octadecenoic acid isomer V	Fatty acids	$C_{18}H_{34}O_5$	329.2335	n.d.	-0.46	n.d.	*329.2338, 312.2277, 293.2139, 229.1458, 211.1362, 183.1427,* ***171.1031***	n.d.	n.d.	*FA 18:1+3O*
178	Other	17.56	Dihydroxyhexadecanoic acid isomer I	Fatty acids	$C_{16}H_{32}O_4$	287.2229	n.d.	-0.41	n.d.	**287.2234**, 269.2132, 223.2114	n.d.	n.d.	*9,16-dihydroxy-palmitic acid OR 10,16-dihydroxy-palmitic acid OR 8,16-dihydroxy-palmitic acid (in silico)*
183	C7	18.17	Trihydroxy-octadecenoic acid isomer VI	Fatty acids	$C_{18}H_{34}O_5$	329.2333	n.d.	0.14	n.d.	**329.2340**, 311.2239, 293.2151, 229.1422, 211.1338, 199.1342, 181.1219	n.d.	n.d.	*9,12,13-Trihydroxyoctadeca-10(E)-dienoic acid (in silico)*
185	C7	18.6	Trihydroxy-octadecenoic acid isomer VII	Fatty acids	$C_{18}H_{34}O_5$	329.2338	n.d.	-1.37	n.d.	**329.2332**, 311.2234, 294.2159, 229.1442, 211.1338, 199.1335, 181.1234	n.d.	*n.d.*	*9,10,13-Trihydroxy-11-octadecenoic acid (in silico)*
187	C2	18.64	Hydroperoxy-octadeca-trienoic acid isomer I	Fatty acids	$C_{18}H_{30}O_4$	309.207	n.d.	0.43	n.d.	**291.1967**, 263.2043, 251.1651, 235.1671, 171.0989	n.d.	*n.d.*	*FA 18:3+2O OR (in silico) 9(S)-hydroperoxy-10(E),12(Z),15(Z)-octadecatrienoic acid*

190	C2		18.89	Hydroperoxy-octadeca-trienoic acid isomer II	Fatty acids	$C_{18}H_{30}O_4$	309.2076	n.d.	-1.51	n.d.	309.2064, **291.1966**, 251.1639, 235.1708, 185.1207, 171.1025	n.d.	*n.d.*	*FA 18:4+2O OR (in silico) 9(S)-hydroperoxy-10(E),12(Z),15(Z)-octadecatrienoic acid*
191	C2	C2	19	Hydroperoxy-octadecadienoic acid isomer I	Fatty acids	$C_{18}H_{32}O_4$	311.2228	295.2270 [M-H$_2$O+H]$^+$	-0.05	-0.73	311.2230, **293.2138**, 275.2012, 263.2008, 171.1019	295.2270, **277.2153**, 259.2046, 195.1380, 135.1171	n.d.	*18:2+2O OR (in silico) 8,13-dihydroxy-9,11-octadecadienoic Acid*
193	Other		19.04	Hydroxy-oxooctadecatrienoic acid isomer I	Fatty acids	$C_{18}H_{28}O_4$	307.1918	n.d.	-1.08	n.d.	235.1351, 209.1179, **185.1184**, 121.0656	n.d.	315	*FA 18:4+2O OR Corchorifatty acid D*
195	C6		19.13	Trihydroxy-octadecenoic acid isomer VIII	Fatty acids	$C_{18}H_{34}O_5$	329.233	n.d.	1.05	n.d.	329.2332, 293.2104, 275.2022, 212.1358, **201.1133**, 171.1024, 139.1121	n.d.	n.d.	*9,10,11-Trihydroxyoctadec-12-enoic acid (in silico)*
199	C2	C2	19.46	Hydroperoxy-octadecadienoic acid isomer II	Fatty acids	$C_{18}H_{32}O_4$	311.2228	277.2158 [M-2×H$_2$O+H]$^+$	-0.05	1.3	**311.2232**, 293.2129, 275.2032, 263.2031, 195.1397	**277.2150**, 199.1514, 121.1005	n.d.	*In silico 13-L-Hydroperoxylinoleic acid OR (9Z,15Z)-12,13-dihydroxyoctadeca-9,15-dienoic acid*
205	Other	C2	20.17	Dioxooctadecatrienoic acid isomer I	Fatty acids	$C_{18}H_{26}O_4$	305.1757	307.19	0.43	1.26	**249.1486**, 205.1626, 135.0772	**289.1798**, 271.1690, 233.1525, 159.0805, 125.0962	n.d.	*FA 18:5+2O = 9,16-Dioxo-10,12,14-octadecatrienoic acid*
211	Other		20.98	Hydroperoxy-octadeca-trienoic acid isomer III	Fatty acids	$C_{18}H_{30}O_4$	309.2064	n.d.	2.36	n.d.	**209.1176**, 207.1398, 185.1183	n.d.	n.d.	*9-HpOTrE OR In silico 13(S)-Hydoperoxylinolenic acid*
214	Other		21.38	Epoxy-oxooctadecenoic acid	Fatty acids	$C_{18}H_{30}O_4$	309.2062	n.d.	3	n.d.	195.1014	n.d.	n.d.	*12(13)Ep-9-KODE (in silico)*
217	C6	Other	21.66	Hydroperoxyoctadecadienoic acid isomer I	Fatty acids	$C_{18}H_{32}O_4$	311.2224	277.2164 [M-2×H$_2$O+H]$^+$	1.23	-0.62	311.2203, 293.2104, 275.2007, **201.1134**	**277.2158**, 235.1714	n.d.	*9(S)-HPODE*

229	C7	C12	22.97	Dihydroxy-octadecenoic acid isomer I	Fatty acids	$C_{18}H_{34}O_4$	313.2385	315.2536	-0.21	-1.95	**313.2386**, 295.2277, 277.2172, 195.1388, 183.1379, 129.0893	**315.2319**, 297.2190, 239.1764, 147.1180, 109.0658	n.d.		*FA 18:1+2O OR (in silico) isoleukotoxin*
231	C2	C2	23.14	Hydroperoxyoctadecadienoic acid isomer II	Fatty acids	$C_{18}H_{32}O_4$	311.2226	295.2271 $[M-H_2O+H]^+$	0.59	-1.05	311.1410, **293.2109**, 201.1127, 185.1185, 171.1010,	295.2266, **277.2161**, 259.2049, 171.1010	n.d.		*9-HPODE*
235	C6	C12	23.38	Dihydroxy-octadecenoic acid isomer II	Fatty acids	$C_{18}H_{34}O_4$	313.2379	315.253	1.7	-0.04	313.2391, 295.2286, 277.2156, **201.1130**	**315.2319**, 297.2190, 239.1764, 147.1180	n.d.		*9,10-DiHOME OR (in silico) leukotoxin*
236	C2		23.58	Hydroperoxyoctadecadienoic acid isomer III	Fatty acids	$C_{18}H_{32}O_4$	311.2225	n.d.	0.91	n.d.	311.1684, 293.2106, 275.2044, 201.1128, **171.1018**	n.d.	n.d.		*9-HPODE*
238		Other	23.72	Capric acid	Fatty acids	$C_{10}H_{20}O_2$	n.d.	173.1533	n.d.	1.78	n.d.	**173.1538**, 153.1271, 131.1065, 103.0748	n.d.	[31]	*In silico*
242	C2		24.46	Hexadecanedioic acid isomer I	Fatty acids	$C_{16}H_{30}O_4$	285.2067	n.d.	1.51	n.d.	285.2064, **267.1964**, 223.2057	n.d.	n.d.		*Hexadecanedioic acid (in silico)*
243	Other		24.57	Octadecatetraenoic acid isomer I	Fatty acids	$C_{18}H_{28}O_2$	275.2014	n.d.	0.92	n.d.	275.2012	n.d.	n.d.		*Stearidonic acid (in silico)*
244	C2	C2	24.64	Hydroxy-octadecatrienoic acid isomer I	Fatty acids	$C_{18}H_{30}O_3$	293.2122	277.216	0.06	0.7	293.2113, **275.2014**, 231.2112, 183.1389, 171.1015	**277.2166**, 149.1327, 135.1169, 121.1013	n.d.		*(9S,10E,12Z,15Z)-9-Hydroxy-10,12,15-octadecatrienoic acid (in silico)*
245	Other		24.66	Octadecatetraenoic acid isomer I	Fatty acids	$C_{18}H_{28}O_2$	275.2009	n.d.	2.73	n.d.	275.1997	n.d.	n.d.		*Stearidonic acid (in silico)*
247	C2		24.81	Hydroxy-octadecatrienoic acid isomer II	Fatty acids	$C_{18}H_{30}O_3$	293.2118	n.d.	1.42	n.d.	**293.2122**, 275.2008, 223.1338, 195.1389	n.d.	n.d.		*13-HOTrE*
248		C9	24.82	1-g-Linolenoyl-glycerol	Fatty acids	$C_{21}H_{36}O_4$	n.d.	353.2682	n.d.	1.24	n.d.	353.2686, 335.2578, 279.2282, **261.2210**,	n.d.		

												243.2109, 233.2248, 205.1586, 163.1475		
253	C2	C2	25.1	Hydroxy-octadecadienoic acid isomer I	Fatty acids	$C_{18}H_{32}O_3$	295.2277	297.242	0.57	1.42	**295.2280**, 277.2172, 193.7454	297.2420, **279.2316**, 261.2204, 243.2095, 223.1687, 179.1423	n.d.	*9-HODE*
254	Other		25.15	Hydroxy-hexadecanoic acid isomer I	Fatty acids	$C_{16}H_{32}O_3$	271.2282		-1.22		**271.2273**, 225.2246		n.d.	*(R)-3-Hydroxy-hexadecanoic acid (in silico)*
256		Other	25.41	2,3-dihydroxypropyl 9-oxooc-tadeca-10,12-dienoate	Fatty acids	$C_{21}H_{36}O_5$	n.d.	351.2525 [M-H$_2$O+H]$^+$	n.d.	1.32	n.d.	351.2545, **235.1686**, 135.0797	n.d.	
40	Other		4.52	Hexosyl hydroxyjasmonic acid	Fatty acyl glycosides	$C_{18}H_{28}O_9$	387.1666	n.d.	-1.4	**n.d.**	**387.1674**, 207.1024, 119.0344	n.d.	n.d.	*12:4+3O fatty acyl hexoside*
67		Other	6.63	Dihydroxy-dimethyldode-cenedioic acid dihexoside	Fatty acyl glycosides	$C_{26}H_{44}O_{16}$	611.2551	613.2703	0.91	-0.14	251.1277, **207.1382**, 189.1287	**289.1640**, 271.1532, 253.1427, 235.1325, 217.1219, 193.1218	n.d.	*in silico*
80	Other		7.99	Hydroxydimethyldecenedioic acid hexoside	Fatty acyl glycosides	$C_{18}H_{30}O_{10}$	405.1761	n.d.	1.28	**n.d.**	**225.1122**, 207.1004, 181.1241	n.d.	n.d.	*in silico*
43		Other	4.66	Riboflavin	Flavins	$C_{17}H_{20}N_4O_6$	n.d.	377.1447	n.d.	2.29	n.d.	**377.1451**, 243.086	n.d.	*Riboflavin*
23		C3	3	Myricetin 3,3'-dihexoside	Flavonoid *O*-glycosides	$C_{27}H_{30}O_{18}$	n.d.	643.148	n.d.	3.88	n.d.	481.0977, **319.0440**, 127.0383		*Flavonol base + 5O, O-Hex, O-Hex*
26		Other	3.16	Cyanidin-3-O-hexoside	Flavonoid *O*-glycosides	$C_{21}H_{20}O_{11}$	n.d.	449.1069	n.d.	2.09	n.d.	449.1068, **287.0541**	n.d.	*Cyanidin-3-O-glucoside OR Kaempferol-3-O-glucoside OR Plantaginin*

27		Other	3.4	Okanin-4'-O-hexoside	Flavonoid *O*-glycosides	$C_{21}H_{22}O_{11}$	n.d.	451.1226	n.d.	1.97	n.d.	449.1079, **289.0698**, 287.0538, 271.0597, 261.0739, 179.0318, 1630382, 139.0382	n.d.		*Marein*
28	Other		3.42	4'-Methylepigallocatechin 5-O-hexoside	Flavonoid *O*-glycosides	$C_{22}H_{26}O_{12}$	481.1348	n.d.	0.73	n.d.	287.0569, **269.0451**, 241.0498, 201.0560, 164.0095, 161.0241, 125.0260	n.d.	n.d.		*4′-Methylepigallocatechin 5-O (In-silico)*
29		C3	3.62	Quercetin-3,4′-*O*-dihexoside	Flavonoid *O*-glycosides	$C_{27}H_{30}O_{17}$	n.d.	627.1534	n.d.	3.48	n.d.	627.148, 465.1021, **303.0492**, 145.0497	n.d.		*Quercetin-3,4′-O-di-beta-glucoside*
35	C1	C3	4.15	Patuletin 3-*O*-dihexoside (Quercetagetin 6-methyl ether 3-*O*-dihexoside)	Flavonoid *O*-glycosides	$C_{28}H_{32}O_{18}$	655.151	657.1656	0.9	0.82	492.0900, **329.0303**, 315.0130, 301.0341, 286.0130, 258.0146	495.1134, **333.0602**, 145.0499	n.d.		*Patuletin 3-gentiobioside (Flavonol base + 4O, 1MeO, O-Hex-Hex)*
41	C4	Other	4.61	Eriodictyol-7-*O*-hexoside isomer I	Flavonoid *O*-glycosides	$C_{21}H_{22}O_{11}$	449.1079	451.1224	2.3	2.42	288.0609, 161.0244, **151.0017**, 135.0432, 125.0229	331.0844, 313.0691, **289.0697**, 163.0368, 153.0163, 145.0499	282		*Eriodictyol-7-O-glucoside*
48	C4		5.06	Eriodictyol-7-*O*-hexoside isomer II	Flavonoid *O*-glycosides	$C_{21}H_{22}O_{11}$	449.1087	451.1236	0.52	0.3	313.0556, 287.0562, 242.0530, 223.0530, **151.0024**, 135.0448	n.d.	285		*Eriodictyol-7-O-glucoside*
50		Other	5.1	Luteolin 7-*O*-malonylhexoside isomer I	Flavonoid *O*-glycosides	$C_{24}H_{22}O_{14}$	n.d.	535.1083	n.d.	-0.13	n.d.	535.1073, 491.1191, 373.0913, **287.0545**	n.d.		*Flavone base + 4O, O-MalonylHex*
54		Other	5.25	Luteolin 7-*O*-malonylhexoside isomer II	Flavonoid *O*-glycosides	$C_{24}H_{22}O_{14}$	n.d.	535.1074	n.d.	1.56	n.d.	535.1082, 373.0852, **287.0544**, 257.0460	n.d.		*Flavone base + 4O, O-MalonylHex*
57	C1	C3	5.4	Quercetagitrin (Quercetagetin 7-*O*-hexoside)	Flavonoid *O*-glycosides	$C_{21}H_{20}O_{13}$	479.0824	481.0974	1.49	0.56	**317.0299**, 316.0220, 299.0196, 287.0188, 271.0237, 194.9931, 165.9902	481.0969, **319.0441**, 145.0497	260, 350	[32]	*Gossypin OR Gossypetin-8-glucoside OR myricetin-3-O-hexoside*

62	C4	Other	6.02	Eriodictyol 7-O-dihexoside	Flavonoid *O*-glycosides	$C_{27}H_{32}O_{16}$	611.1618		-0.07		287.0569, 210.5165, 135.0446		n.d.		*in silico (Eriodictyol 7-O-sophoroside)*
69	C1	C3	6.86	Hyperoside OR **Isoquercitrin** (Quercetin 3-*O*-hexoside)	Flavonoid *O*-glycosides	$C_{21}H_{20}O_{12}$	463.0877	465.1025	1.08	**0.54**	**300.0266**, 271.0241, 255.0292, 243.0288, 178.9984	**303.0498**, 145.0486	257, 355	[33]	*Quercetin-3-O-glucoside*
70	C1	C3	6.96	Quercetagetin 6-methylether 3-*O*-hexoside	Flavonoid *O*-glycosides	$C_{22}H_{22}O_{13}$	493.099	495.114	-0.48	**-1.38**	**330.0372**, 315.0142, 287.0192, 271.0251, 243.0292, 164.9808	**333.0604**, 145.0502	280, 343		*Flavonol base + 4O, 1MeO, O-Hex OR NCGC00385532-01!2-(3,5-dihydroxy-4-methoxyphenyl)-5,7-dihydroxy-3-[(2S,3R,4S,5S,6R)-3,4,5-trihydroxy-6-(hydroxymethyl)oxan-2-yl]oxychromen-4-one*
75	C1	C3	7.59	Patulitrin (Patuletin -7-*O*-hexoside)	Flavonoid *O*-glycosides	$C_{22}H_{22}O_{13}$	493.0978	989.2176 $[2M+H]^+$	1.95	1.22	479.0750, 331.0448, 330.0376, **315.0143**, 312.0267, 299.0239, 287.0191, 271.0249, 259.0241, 243.0293, 181.0149	495.1132, **333.0603**	260, 365	[32]	*NCGC00385532-01!2-(3,5-dihydroxy-4-methoxyphenyl)-5,7-dihydroxy-3-[(2S,3R,4S,5S,6R)-3,4,5-trihydroxy-6-(hydroxymethyl)oxan-2-yl]oxychromen-4-one*
76	C1	C3	7.61	Quercetin 3-O-(malonyl-hexoside)	Flavonoid *O*-glycosides	$C_{24}H_{22}O_{15}$	505.0978 $[M-CO_2-H]^-$	551.103	1.75	0.27	**300.0284**, 271.0267, 255.0296	551.1046, **303.0501**, 231.0496, 159.0288, 145.0495, 127.0391, 109.0280	260, 365		*Flavonol base + 4O, O-MalonylHex*
84	Other	Other	8.24	Patuletin 3-*O*-deoxyhexoside	Flavonoid *O*-glycosides	$C_{22}H_{22}O_{12}$	477.1033	479.118	1.15	0.84	330.0117, 314.0428, **299.0191**, 271.0251,	**333.0604**, 317.0656, 129.0540	270, 340		*Petunidin-3-O-beta-glucopyranoside*

											243.0297, 215.0321, 164.9848				
86	C1	C3	8.3	Quercetagetin 8-methylether 7-*O*-hexoside	Flavonoid *O*-glycosides	$C_{22}H_{22}O_{13}$	493.0975	495.1133	2.56	0.03	331.0454, **316.0217**, 303.0522, 287.0187, 271.0258, 257.0098, 243.0270, 181.0139, 165.9897	**495.1135**, 375.0707, 333.0606, 303.0499	270, 340		*Flavonol base + 4O, O-Hex, 1MeO*
91	C4		8.75	Eriodictyol-7-*O*-hexoside isomer III	Flavonoid *O*-glycosides	$C_{21}H_{22}O_{11}$	449.1081	n.d.	1.86	n.d.	449.1054, **287.0555**, 269.0447, 151.0025, 135.0435	n.d.	284		*Eriodictyol-7-O-glucoside*
100	C1	C3	9.98	Eupatolitin 3-*O*-pentosyl-hexoside	Flavonoid *O*-glycosides	$C_{28}H_{32}O_{17}$	639.1556	641.1718	1.68	-0.9	344.0534, **329.0299**, 314.0071, 301.365, 286.0127	509.1292, **347.0764**, 133.0496	n.d.		*In silico*
101	C1	C3	10.48	Eupatolitin 3-*O*-hexoside	Flavonoid *O*-glycosides	$C_{23}H_{24}O_{13}$	507.1138	509.1302	4	-2.43	344.0533, **329.0299**, 314.0068, 301.0351, 286.0118, 270.0162, 258.0168	**347.0770**, 145.0499	260, 350		*Syringetin-3-O-galactoside*
104	C1	Other	10.92	Helichrysoside (Quercetin 3-*O*-(*p*-coumaroylhexoside))	Flavonoid *O*-glycosides	$C_{30}H_{26}O_{14}$	609.1249	611.1401	0.13	-0.93	463.0890, **300.0279**, 271.0249, 255.0292, 243.0311, 227.0350, 178.9991, 151.0044	309.0974, 303.0504, 291.0867, 165.055, **147.0445**, 119.0496	315		*3-Glu-7-Rha Quercetin*
105	C1	C3	10.97	Patuletin 3-*O*-(acetyl-deoxy-hexoside)	Flavonoid *O*-glycosides	$C_{24}H_{24}O_{13}$	519.1149	521.1302	-0.93	-2.37	330.0385, **315.0148**, 287.0192, 271.0250, 243.0306,	**333.0611**, 189.0755, 129.0552, 111.0445	n.d.	[34]	*[2-(3,4-dihydroxyphenyl)-5-hydroxy-7-methoxy-4-oxo-3-[(2S,3R,4R,5R,6S)-3,4,5-tri-hydroxy-6-methyloxan-2-yl]oxychromen-8-yl] acetate OR Mearnsetin 3-O-(4′′-O-*

															acetyl)-alpha-L-rhamnopyranoside (in silico)
108	C1		11.38	Quercetin 3-*O*-(acetyl-deoxyhexoside)	Flavonoid *O*-glycosides	$C_{23}H_{22}O_{12}$	489.1033	n.d.	1.12	n.d.	**300.0278**, 271.0248, 255.0309, 243.0291	n.d.	n.d.		*Quercetin 3-(2′′-acetylrhamnoside) (in silico)*
117	C1	C3	11.77	Eupalitin 3-*O*-hexoside	Flavonoid *O*-glycosides	$C_{23}H_{24}O_{12}$	491.1194	493.1349	0.2	-1.72	328.0591, **313.0352**, 299.0191, 285.0400, 270.0170	493.1355, **331.0820**	269, 335		*Malvidin-3-O-glucoside*
121	C1		12.13	Eupatolin (Eupatolitin-3-*O*-deoxyhexoside)	Flavonoid *O*-glycosides	$C_{23}H_{24}O_{12}$	491.1192	493.1353	0.61	-2.54	344.0536, **329.0299**, 315.0139, 301.0355, 286.0115	347.0767	260, 340	[32]	*In silico*
122		Other	12.15	Jacein (Jaceidin-7-*O*-hexoside) isomer I	Flavonoid *O*-glycosides	$C_{24}H_{26}O_{13}$	n.d.	523.1448	n.d.	-0.35	n.d.	523.1459, **361.0924**	n.d.		*In silico*
124	C1	C3	12.26	Eupatolitin-3-*O*-(acetyl-hexoside) isomer I	Flavonoid *O*-glycosides	$C_{25}H_{26}O_{14}$	549.1248	551.1404	0.33	-1.58	344.0541, **329.0302**, 314.0073, 301.0355, 286.0128, 269.0103	551.1403, **347.0765**, 187.0602	344		*Syringetin 3-(6′′-acetylglucoside) (in silico)*
125	C1	C3	12.41	Jacein [5,7,4′-trihydroxy-3,6,3′-trimethoxyflavone-7-*O*-hexoside] isomer II	Flavonoid *O*-glycosides	$C_{24}H_{26}O_{13}$	521.1304	523.1453	-0.64	-1.31	359.0774, 344.0536, **329.0307**, 314.0071, 301.0357, 286.0116	523.1452, **361.0922**	260, 345	[35]	*Centaurein OR Jacein (in silico)*
127		Other	12.56	Eupalitin-3-*O*-malonyl-hexoside	Flavonoid *O*-glycosides	$C_{26}H_{26}O_{15}$	n.d.	579.1346	n.d.	-0.26	n.d.	579.1332, **331.0813**	n.d.		*in silico*
128		C3	12.6	Eupatolitin-3-*O*-(acetyl-hexoside) isomer II	Flavonoid *O*-glycosides	$C_{25}H_{26}O_{14}$	n.d.	551.1393	n.d.	0.42	n.d.	551.1379, **347.0764**, 145.0494			*in silico*
133		C3	13.05	Jaceidin-7-*O*-(hydroxymethyl-glutaryl-hexoside)	Flavonoid *O*-glycosides	$C_{30}H_{34}O_{17}$	n.d.	667.1859	n.d.	1.46	n.d.	667.1844, **361.0913**, 145.0491	n.d.		*in silico*

134	C1	C3	13.09	Jaceidin-7-*O*-(malonyl-hexoside)	Flavonoid *O*-glycosides	$C_{27}H_{28}O_{16}$	563.1400 [M-CO₂-H]⁻	609.1438	1.03	1.99	344.0531, **329.0300**, 314.0082, 301.0337, 287.0137	609.1456, **361.0916**, 127.0387	n.d.		*in silico*
138	C1	C3	13.72	6,7-Dimethoxy-3,5,4'-trihydroxyflavone-3-*O*-deoxyhexoside (Eupalitin-3-*O*-deoxyhexoside)	Flavonoid *O*-glycosides	$C_{23}H_{24}O_{11}$	475.1242	477.1387	0.81	0.92	328.0585, **313.0353**, 299.0195, 286.0484, 271.0245	**331.0808**, 129.0541	269, 330	[32]	*in silico*
141	C1		14.02	Jaceidin-7-*O*-deoxyhexoside	Flavonoid *O*-glycosides	$C_{24}H_{26}O_{12}$	505.1346	n.d.	1.09	n.d.	359.0763, **343.0470**, 328.0208, 301.0333, 285.0025	n.d.	n.d.		*Jaceidin 7-rhamnoside (in silico)*
142	C1	C3	14.05	Eupatolitin-3-*O*-(acetyl-deoxyhexoside) isomer I	Flavonoid *O*-glycosides	$C_{25}H_{26}O_{13}$	533.1303	535.144	-0.44	1.16	344.0536, **329.0300**, 314.0068, 301.0354, 286.0119, 270.0165, 258.0165	**347.0761**, 189.0754, 171.0649, 129.0543, 111.0437	260, 340	[32]	
152	C1	C3	14.93	Eupatolitin-3-*O*-(Ac-Dhex) isomer II	Flavonoid *O*-glycosides	$C_{25}H_{26}O_{13}$	533.1299	535.1443	0.31	0.59	344.0537, **329.0301**, 315.0141, 301.0355, 286.0118, 270.0165, 258.0165	**347.0757**, 189.0749, 129.0539	260, 340	[32]	
153	C1	C3	15.07	Eupatolitin-3-*O*-(Ac-Dhex) isomer III	Flavonoid *O*-glycosides	$C_{25}H_{26}O_{13}$	533.1297	557.1254 [M+Na]⁺	0.68	0.97	344.0536, **329.0301**, 314.0066, 301.0350, 286.0116, 270.0171, 258.0169	**557.1243**, 369.0582, 211.0565, 171.0618, 129.0545	261, 340	[32]	
170		C3	16.73	Eupalitin-3-*O*-acetyl-deoxyhexoside	Flavonoid *O*-glycosides	$C_{25}H_{26}O_{12}$	n.d.	519.1497	n.d.	0.01	n.d.	**331.0810**, 129.0538	n.d.	[32]	*In silico*
102	Other	Other	10.76	Quercetin	Flavonols	$C_{15}H_{10}O_7$	301.0358	303.0504	-1.4	**-1.56**	**301.0359**, 273.0401, 245.0465, 229.0500, 178.9995, 151.0017	**303.0505**, 229.0467, 195.0271	370	[36]	*Quercetin*

145	C1	Other	14.26	**Eupatolitin** (3,3',4',5-tetrahydroxy-6,7-dimethoxyflavone)	Flavonols	$C_{17}H_{14}O_8$	345.0613	347.0755	0.84	1.86	345.0608, 330.0386, **315.0148**, 287.0201, 271.0246	**347.0752**, 332.0514	n.d.	[32]	*Gossypetin 3,8-dimethyl ether (in silico)*
175		Other	17.42	Chrysosplenol D	Flavonols	$C_{18}H_{16}O_8$	n.d.	361.0913	n.d.	1.37	n.d.	**361.0910**, 346.0668, 328.0588, 300.0620, 272.0609	n.d.	[37]	
194		Other	19.12	Hexamethylquercetagetin	Flavonols	$C_{21}H_{22}O_8$	n.d.	403.1386	n.d.	0.36	n.d.	**403.1381**, 388.1131, 373.0920, 342.1104		[38]	*In silico*
208		Other	20.6	Tangeritin	Flavonols	$C_{20}H_{20}O_7$	n.d.	373.1283	n.d.	-0.32	n.d.	373.1280, 358.1041, 343.0822	n.d.	[39]	*Tangeritin*
251	Other	C9	25.03	Gingerglycolipid A	Glycosylmonoacylglycerols	$C_{33}H_{56}O_{14}$	721.3644 $[M+FA-H]^-$	515.3216 $[M-Hex+H]^+$	1.2	-0.27	**277.2166**, 235.0810, 185.0464	497.3100, 353.2684, **261.2207**, 149.1332	n.d.		*DGMG 18:3*
255		C9	25.16	Unidentified glycosyl-monoacylglycerol	Glycosylmonoacylglycerols	$C_{30}H_{42}O_{11}$	n.d.	579.2828	n.d.	-4.86	n.d.	579.2825, 561.2715, **353.2681**, 335.2573, 279.2298, 261.2209, 243.2100	n.d.		
10	Other		1.41	*p*-Coumaroyl-(hexosyl)hexose	Hydroxycinnamic acid glycosides	$C_{21}H_{28}O_{13}$	533.15125 $[M+FA-H]^-$	n.d.	-0.01	n.d.	325.0944, 205.0521, 163.0369, **145.0284**, 119.0496	n.d.	n.d.		*NCGC00385695-01_C21H28O13_alpha-D-Glucopyranoside, beta-L-fructofuranosyl 6-O-[(2E)-3-(4-hydroxyphenyl)-1-oxo-2-propen-1-yl]-*
13	C4		1.83	Caffeoyl-hexose	Hydroxycinnamic acid glycosides	$C_{15}H_{18}O_9$	341.0879	n.d.	-0.28	n.d.	**179.0352**, 135.0436	n.d.	n.d.		*Caffeic acid hexoside*
14	C4		1.93	*p*-Coumaroyl-hexose isomer I	Hydroxycinnamic acid glycosides	$C_{15}H_{18}O_8$	371.09799 $[M+FA-H]^-$	327.1069	1.13	1.67	**163.0391**, 119.0493	n.d.	n.d.		*NCGC00180738-02!(E)-3-[2-[(2S,3R,4S,5S,6R)-3,4,5-trihydroxy-6-*

															(hydroxymethyl)oxan-2-yl]oxyphenyl]prop-2-enoic acid
22	Other		2.98	*p*-Coumaric acid hexoside	Hydroxycinnamic acid glycosides	$C_{15}H_{18}O_8$	325.0931	n.d.	-0.64	n.d.	234.9707, **145.0284**	n.d.	n.d.		*Coumaroyl Hexoside (isomer of 690, 691)*
25	C4		3.14	*p*-Coumaroyl-hexose isomer II	Hydroxycinnamic acid glycosides	$C_{15}H_{18}O_8$	325.0931	344.1331 $[M+NH_4]^+$	-0.33	2.74	289.0092, 163.0397, **119.0491**	309.0971, **165.0538**, 147.0440, 127.0380	n.d.		*Coumaroyl Hexoside (isomer of 691, 692)*
39	C3	Other	4.41	Coumaroyl-glucuronosylglycerol	Hydroxycinnamic acid glycosides	$C_{18}H_{22}O_{11}$	413.1083	415.1224	1.53	**2.63**	**267.0718**, 249.0604, 237.0776, 163.0357, 119.0494, 113.0245	397.1108, 323.0745, 239.0904, **147.0437**, 119.0506	n.d.	[40,41]	*in silico (based on similar compound)*
56	C3		5.32	Feruloyl-glucuronosylglycerol	Hydroxycinnamic acid glycosides	$C_{19}H_{24}O_{12}$	443.1192	n.d.	0.68	n.d.	295.08365, **267.0721**, 249.064, 193.0494, 175.0404, 134.0369	n.d.	n.d.	[40,41]	*in silico*
21	Other		2.94	Caffeic acid	Hydroxycinnamic acids	$C_9H_8O_4$	179.0352	181.0485	-1.21	5.75	179.0352, **135.0439**	163.0382, 145.0281, 135.0440, 120.0800	320		*Caffeic acid*
37	Other	Other	4.3	*p*-Coumaric acid	Hydroxycinnamic acids	$C_9H_8O_3$	163.0394	165.0539	4.07	4.39	163.0391, **119.0491**	165.0535, **147.0435**, 119.0486, 91.0528	310		*3-Hydroxycinnamic acid*
47	Other		5.03	Hydroxyjasmonic acid	Jasmonic acids	$C_{12}H_{18}O_4$	225.1129		1.47		225.1124		n.d.		*(-)-12-hydroxyjasmonic acid*
250		Other	25.02	Viriditin	N-acylpyrrolidines	$C_{18}H_{29}NO_3$	n.d.	308.2214	n.d.	2.02	n.d.	308.2221, 290.2111, **192.1377**, 164.1063, 125.0954	n.d.		
46	Other		4.96	Benzoyl(hexuronosyl)-glycerol	*O*-glucuronides	$C_{16}H_{20}O_{10}$	371.0977	n.d.	1.8	n.d.	249.0592	n.d.	n.d.		*(2S,3S,4S,5R,6R)-6-(3-benzoyloxy-2-hydroxypropoxy)-3,4,5-trihydroxyoxane-2-carboxylic acid*
18	Other		2.65	Dihydrocoumaroyl hexoside	Phenolic glycosides	$C_{15}H_{20}O_8$	327.10944	n.d.	-2.62	n.d.	**165.0573**	n.d.			*Dihydrocoumaroyl Hexoside*

233		C12	23.25	Progesterone	Pregnane steroids	$C_{21}H_{30}O_2$	n.d.	315.2319	n.d.	-0.14	n.d.	**315.2319**, 297.2190, 239.1764, 147.1180, 109.0658	n.d.	*Progesterone*
227		Other	22.8	Unidentified hexoside	Pregnane steroids?	$C_{24}H_{30}O_6$	n.d.	432.2389 $[M+NH_4]^+$	n.d.	-2.02	n.d.	415.2119, 281.1391, 147.0655, 135.0806, **119.0858**, 107.0851	n.d.	
3		Other	0.77	Adenosine	Purine nucleosides	$C_{10}H_{13}N_5O_4$	n.d.	268.1038	n.d.	0.86	n.d.	268.1035, **136.0614**	n.d.	*Adenosine*
6		Other	0.94	Methyladenosine	Purine nucleosides	$C_{11}H_{15}N_5O_4$	n.d.	282.1185	n.d.	4.2	n.d.	282.1192, **136.0613**	n.d.	*2-O-Methyladenosine*
1	Other		0.62	Quinic acid	Quinic acids and derivatives	$C_7H_{12}O_6$	191.0559	n.d.	1.1	n.d.	**191.0557**, 173.0438, 127.0399	n.d.	n.d.	*D-(-)-quinic acid*
12	C3	C6	1.78	Neochlorogenic acid (3-CQA)	Quinic acids and derivatives	$C_{16}H_{18}O_9$	353.0879	355.1022	-0.27	0.45	**191.0563**, 179.0354, 135.0433	**163.0381**, 145.0286, 135.0436, 117.0328	244, 320	*Neochlorogenic acid*
17	C3		2.6	*trans*-3-*O*-*p*-Coumaroyl quinic acid (*trans*-3-*p*CoQA)	Quinic acids and derivatives	$C_{16}H_{18}O_8$	337.0927	n.d.	0.57	n.d.	191.0545, **163.0391**, 119.0498	n.d.	n.d.	*Coumaroyl quinic acid (isomer of 759, 760) OR 3-p-Coumaroylquinic acid*
16	C3		2.5	*cis*-3-*O*-*p*-Coumaroyl quinic acid (*cis*-3-*p*CoQA)	Quinic acids and derivatives	$C_{16}H_{18}O_8$	337.0931	n.d.	-0.62	n.d.	**191.0573**, 163.0400, 155.0364, 119.0491	n.d.	n.d.	*Coumaroyl quinic acid (isomer of 758, 759)*
19	C3	C6	2.77	Chlorogenic acid (5-CQA)	Quinic acids and derivatives	$C_{16}H_{18}O_9$	353.088	355.1013	-0.55	2.99	191.0562	337.0909, **163.0381**, 145.0279, 135.0436	244, 325	*Chlorogenic acid*
24	C3		3.09	Cryptochlorogenic acid (4-CQA)	Quinic acids and derivatives	$C_{16}H_{18}O_9$	353.0878	355.1018	0.02	**1.58**	**191.0565**, 179.0353, 173.0440, 135.0444, 127.0427	n.d.	320	*Caffeoylquinic acid OR 4-Caffeoylquinic acid*
32	C3	Other	3.93	*trans*-5-*O*-*p*-Coumaroyl quinic acid (*trans*-5-*p*CoQA)	Quinic acids and derivatives	$C_{16}H_{18}O_8$	337.0926	339.1071	0.86	**1.01**	**191.0560**, 173.0437, 163.0382, 127.0421, 119.0512, 111.0444	165.0528, **147.0434**, 119.0484	310	*Coumaroyl quinic acid (isomer of 758, 760)*
36	Other		4.16	*trans*-4-*O*-*p*-Coumaroyl quinic acid (*trans*-4-*p*CoQA)	Quinic acids and derivatives	$C_{16}H_{18}O_8$	337.0934	n.d.	-1.5	**n.d.**	**173.0445**, 163.0346, 81.7734	n.d.	n.d.	

44	C3		4.91	5-*O*-Feruloyl quinic acid (5-FQA)	Quinic acids and derivatives	$C_{17}H_{20}O_9$	367.1028	n.d.	1.78	n.d.	193.0500, **191.0561**	n.d.	n.d.		*Feruloyl quinic acid (isomer of 886, 887)*
55	C3		5.28	*cis*-5-*O*-*p*-Coumaroyl quinic acid (*cis*-5-*p*CoQA)	Quinic acids and derivatives	$C_{16}H_{18}O_8$	337.093	n.d.	-0.32	n.d.	**191.0560**, 173.0429, 117.1345	n.d.	n.d.		*Coumaroyl quinic acid (isomer of 758, 759)*
81	C3	C6	8.08	1,3-*O*-Dicaffeoylquinic acid (1,3-DiCQA)	Quinic acids and derivatives	$C_{25}H_{24}O_{12}$	515.1185	517.1342	1.94	-0.29	353.0884, **191.0560**, 179.0346, 173.0445, 135.0431	499.1239, 319.0812, **163.0389**, 145.0284, 135.0443, 117.0335	325	[42]	*Cynarine*
95	C3	C6	9.17	1,5-*O*-Dicaffeoylquinic acid (1,5-diCQA)	Quinic acids and derivatives	$C_{25}H_{24}O_{12}$	515.118	517.1344	2.91	-0.67	353.0879, 191.0555, 179.0345, **173.0447**, 161.0250, 155.0326, 135.0430	499.1233, 337.0910, **163.0391**, 145.0290, 135.0443	325	[42,43]	4,5-diCQA
11	Other	Other	1.44	Pantothenic acid	Secondary alcohols	$C_9H_{17}NO_5$	218.1037	220.1177	-1.39	1.14	146.0814	**220.1177**, 202.1068, 184.0958, 174.1135, 142.0848, 136.0611, 124.0751, 119.0364	n.d.		*Pantothenate*
140		C2	13.97	Jasmolon	Secondary alcohols	$C_{11}H_{16}O_2$	n.d.	181.1215	n.d.	4.48	n.d.	**181.1216**, 163.1110, 135.1163, 107.0846	n.d.		
45	Other	C1	4.94	Rudbeckin A	Sesquiterpene lactones	$C_{15}H_{24}O_5$	283.1546	285.1688	1.75	**2.99**	**283.1543**, 265.1408, 223.1324, 203.1415, 169.1239	267.1592, **249.1480**, 231.1376, 219.1374, 203.1428, 185.1321	n.d.	[25]	
52		C1	5.18	4- OR 15-acetoxyrudmollin	Sesquiterpene lactones	$C_{17}H_{24}O_5$	n.d.	309.1691	n.d.	1.79	n.d.	309.1711, **249.1475**, 203.1420, 175.1109			*in silico*
61		C1	5.91	Acetyl-rudbeckin A isomer I	Sesquiterpene lactones	$C_{17}H_{26}O_6$	n.d.	344.2061 $[M+NH_4]^+$	n.d.	2.04	n.d.	327.1807, 309.1679, 267.1584, **249.1472**, 219.1382, 203.1429	n.d.		*11α,13-Dihydrochamissonolide (in silico)*
74	Other	C1	7.51	2-methyl-2-butenoyl rudbeckin A hexoside	Sesquiterpene lactones	$C_{26}H_{40}O_{11}$	573.2544 $[M+FA-H]^-$	546.2913 $[M+NH_4]^+$	1.64		428.5026	529.2637, **367.2116**, 267.1590, 249.1487,	n.d.		

												231.1381, 219.1382, 173.1318		
79		Other	7.84	Butanoic acid, 2-methyl-, 2,3,3a,4,5,6,7,8,9,11a-decahydro-6,9-dihydroxy-6,10-dimethyl-3-methylene-2-oxocyclodeca[*b*]furan-4-yl ester	Sesquiterpene lactones	$C_{20}H_{30}O_6$	n.d.	367.2115	n.d.	0.04	n.d.	267.1586, 249.1496, 237.1499, **221.1534**, 203.1426		*in silico, note: Isomer of methylbutyl-rudbeckin A*
85		C1	8.27	Unidentified sesquiterpene lactone	Sesquiterpene lactones	$C_{20}H_{32}O_6$	n.d.	369.2273	n.d.	-0.37	n.d.	352.0822, 267.1601, **249.1481**, 219.1387, 203.1429		
89	Other		8.65	Diacetyl-rudbeckin A hexoside isomer I	Sesquiterpene lactones	$C_{26}H_{42}O_{11}$	575.2698 [M+FA-H]$^-$	548.3064 [M+NH$_4$]$^+$	2.1	0.26	n.d.	548.3021, **369.2271**, 351.2168, 267.1593, 249.1480, 237.1476, 219.1378	n.d.	*in silico*
90		C1	8.73	Diacetyl-rudbeckin A hexoside isomer II	Sesquiterpene lactones	$C_{26}H_{42}O_{11}$	575.2696 [M+FA-H]$^-$	548.3066 [M+NH$_4$]$^+$	2.48	-0.12	n.d.	548.3052, 504.0302, **369.2275**, 351.2164, 267.1590, 249.1482, 237.1482, 219.1385	n.d.	*in silico*
93		C1	9.09	Cichorioside C/M OR Lactuside B	Sesquiterpene lactones	$C_{21}H_{32}O_9$	n.d.	446.2385 [M+NH$_4$]$^+$	n.d.	-0.1	n.d.	446.2393, 267.1596**,** **249.1491**, 237.1485, 231.1378, 219.1378		*in silico*
94		C1	9.1	Diacetyl-rudbeckin A isomer I	Sesquiterpene lactones	$C_{19}H_{28}O_7$	n.d.	386.2175 [M+NH$_4$]$^+$	n.d.	-0.47	n.d.	386.2184, 369.1923, 327.1812, **309.1706**, 291.1601, 263.1643, 249.1483, 231.1376, 203.1432	n.d.	*In silico*

99		C1	9.75	2-Butenoic acid, 2-methyl-, 2,3,3a,4,5,6,7,8,9,11a-decahydro-6,7,9-trihydroxy-6,10-dimethyl-3-methylene-2-oxocyclodeca[*b*]furan-4-yl ester	Sesquiterpene lactones	$C_{20}H_{28}O_7$	n.d.	398.2176 $[M+NH_4]^+$	n.d.	-0.71	n.d.	**398.2172**, 381.1916, 363.1819, 281.1389, 263.1271, 251.1280, 233.1176	n.d.	*In silico*
103	Other	C1	10.77	Methylbutenoyl rudbeckin A	Sesquiterpene lactones	$C_{20}H_{30}O_6$	n.d.	384.2388 $[M+NH_4]^+$	n.d.	-2.01	n.d.	384.2408, **367.2127**, 349.2044, 267.1594, 249.1492, 237.1484, 231.1384, 219.1382, 203.1431, 185.1331	n.d.	*In silico*
137		C1	13.45	Diacetyl-rudbeckin A isomer II	Sesquiterpene lactones	$C_{19}H_{28}O_7$	n.d.	386.2166 $[M+NH_4]^+$	n.d.	1.98	n.d.	369.2166, 351.1794, 327.1819, 309.1676, 291.1585, 263.1643, **249.1476**, 231.1379, 219.1370, 203.1420, 185.1318, 175.1106, 157.1004	n.d.	
139		C1	13.91	Acetyl-isobutyl-rudbeckin A	Sesquiterpene lactones	$C_{21}H_{32}O_7$	n.d.	414.2480 $[M+NH_4]^+$	n.d.	1.59	n.d.	397.2219, 355.2132, 337.1994, 309.1685, 291.1592, 263.1637, **249.1471**, 231.1371, 219.1365, 203.1418, 185.1324	n.d.	
146		C1	14.5	Diacetyl-rudbeckin A isomer III	Sesquiterpene lactones	$C_{19}H_{28}O_7$	n.d.	386.2170 $[M+NH_4]^+$	n.d.	0.89	n.d.	369.2169, 351.1793, 285.1700, 267.1574, **249.1477**, 237.1483, 231.1373, 219.1367,	n.d.	

											203.1427, 185.1309, 175.1106		
151	C1	14.76	Acetyl-propionyl dehydro-rudbeckin A	Sesquiterpene lactones	$C_{20}H_{28}O_7$	n.d.	398.2172 $[M+NH_4]^+$	n.d.	0.34	n.d.	381.1922, 307.1523, **265.1438**, 247.1323, 229.1197, 201.1277, 187.1100	n.d.	*in silico*
159	C1	15.47	Acetyl-(2-methyl-2-butenoyl)-rudbeckin A isomer I	Sesquiterpene lactones	$C_{22}H_{32}O_7$	n.d.	426.2476 $[M+NH_4]^+$	n.d.	2.52	n.d.	409.2209, 381.2110, 367.2109, 309.1686, 291.1583, 263.1639, **249.1479**, 231.1370, 219.1375, 203.1418, 185.1319, 175.1107	n.d.	*In silico*
160	C1	15.51	Acetyl-propionyl rudbeckin A	Sesquiterpene lactones	$C_{20}H_{30}O_7$	n.d.	400.2320 $[M+NH_4]^+$	n.d.	2.56	n.d.	400.2316, 383.2058, 365.1948, 309.1690, 267.1568, **249.1473**, 231.1373, 219.1368, 203.1415, 185.1310, 157.1002	n.d.	*In silico*
163	C1	15.9	Acetyl-(2-methyl-2-butenoyl)-rudbeckin A isomer II	Sesquiterpene lactones	$C_{22}H_{32}O_7$	n.d.	426.2478 $[M+NH_4]^+$	n.d.	2.03	n.d.	409.2209, 381.2110, 367.2109, 309.1686, 291.1583, 263.1639, **249.1479**, 231.1370, 219.1375, 203.1418, 185.1319, 175.1107	n.d.	*In silico*

88	Other	C1	8.54	[(10E)-6,9-dihydroxy-6,10-dimethyl-3-methylidene-2-oxo-4,5,7,8,9,11a-hexahydro-3aH-cyclodeca[b]furan-4-yl] (E)-2-methylbut-2-enoate hexoside	Sesquiterpene lactones	$C_{26}H_{38}O_{11}$	571.2395 $[M+FA-H]^-$	544.2758 $[M+NH_4]^+$	0.22	-1.07	n.d.	544.2753, **365.1967**, 265.1430, 247.1331, 219.1376	n.d.	[44]	*in silico*
106		C1	11.06	[(10E)-6,9-dihydroxy-6,10-dimethyl-3-methylidene-2-oxo-4,5,7,8,9,11a-hexahydro-3aH-cyclodeca[b]furan-4-yl] (E)-2-methylbut-2-enoate isomer I	Sesquiterpene lactones	$C_{20}H_{28}O_6$	n.d.	382.2233 $[M+NH_4]^+$	n.d.	-2.43	n.d.	382.2229, 365.1951, 347.1898, **265.1440**, 247.1331, 229.1229, 219.1385, 201.1267	n.d.	[44]	
109		C1	11.41	Argophyllin C OR Euperfolin OR [(10E)-6,9-dihydroxy-6,10-dimethyl-3-methylidene-2-oxo-4,5,7,8,9,11a-hexahydro-3aH-cyclodeca[b]furan-4-yl] (E)-2-methylbut-2-enoate isomer II	Sesquiterpene lactones	$C_{20}H_{28}O_6$	n.d.	365.197	n.d.	-3.11	n.d.	265.1442, 247.1328, 235.1329, **219.1385**, 189.1283, 147.0789	n.d.		*In silico*
110		C1	11.41	Deoxyarctolide	Sesquiterpene lactones	$C_{20}H_{30}O_5$	n.d.	351.2175	n.d.	-2.57	n.d.	351.2176, 305.2122, **249.1492**, 231.1384, 203.1435, 185.1329, 175.1127, 145.1018	n.d.		*In silico*
111	Other	C1	11.42	Unidentified sesquiterpene (methyl-butylated)	Sesquiterpene lactones	$C_{20}H_{32}O_6$	n.d.	369.2281	n.d.	-2.54	n.d.	267.1597, **249.1489**, 231.1384, 219.1384, 203.1433	n.d.		*In silico*
112		C1	11.53	Unidentified sesquiterpene (acetylated)	Sesquiterpene lactones	$C_{20}H_{30}O_7$	n.d.	400.2341 $[M+NH_4]^+$	n.d.	-2.93	n.d.	400.2341, 383.2070, 341.1974, 323.1862, 309.1701, **249.1486**,	n.d.		*In silico*

											231.1388, 203.1432, 157.1027			
114	C1	11.64	Desacylligulatin C OR Rudmollin	Sesquiterpene lactones	$C_{15}H_{22}O_4$	n.d.	284.1861 $[M+NH_4]^+$	n.d.	-1.75	n.d.	284.1860, 267.1588, 249.1491, **237.1495**, 231.1391, 221.1550, 203.1438, 191.1427	n.d.	[25,26]	*In silico*
116	C1	11.75	Zinangustolide isomer I (methyl-butylated)	Sesquiterpene lactones	$C_{20}H_{30}O_6$	n.d.	384.2388 $[M+NH_4]^+$	n.d.	-3.1	n.d.	384.2392, 265.1435, 247.1337, **219.1381**, 201.1265	n.d.		
119	C1	11.83	Methylbutyryl rudbeckin A isomer I	Sesquiterpene lactones	$C_{20}H_{32}O_6$	n.d.	386.2543 $[M+NH_4]^+$	n.d.	-1.59	n.d.	369.2276, 351.2169, 285.1692, 267.1596, **249.1491**, 237.1484, 231.1378, 219.1380, 203.1429, 185.1322	n.d.		*In silico*
120	C1	12.1	Acetyl-rudbeckin A isomer II	Sesquiterpene lactones	$C_{17}H_{26}O_6$	n.d.	344.2072 $[M+NH_4]^+$	n.d.	-1.34	n.d.	327.1809, 317.0667, 267.1596, 249.1484, 237.1485, **219.1383**, 203.1432, 185.1326	n.d.		*In silico*
123	C1	12.2	Zinangustolide isomer II (methyl-butylated)	Sesquiterpene lactones	$C_{20}H_{30}O_6$	n.d.	384.2385 $[M+NH_4]^+$	n.d.	-1.19	n.d.	384.2385, 265.1437, 247.1337, **219.1384**, 201.1272	n.d.		
144	C1	14.24	Methylbutyryl rudbeckin A isomer II	Sesquiterpene lactones	$C_{20}H_{32}O_6$	n.d.	386.2531 $[M+NH_4]^+$	n.d.	1.67	n.d.	369.2261, 351.2146, 305.2108, 267.1582, **249.1473**, 237.1485, 231.1370, 219.1365, 203.1421, 175.1108	n.d.		
147	C1	14.59	Dihydro-zinangustolide isomer I	Sesquiterpene lactones	$C_{20}H_{32}O_6$	n.d.	369.2264	n.d.	2.08	n.d.	351.2152, 285.1682, 267.1588, **249.1479**,	n.d.		*In silico*

											237.1475, 231.1374, 219.1380, 203.1417, 185.1323			
166	C1	16.17	Scapiformolactone D/E	Sesquiterpene lactones	$C_{22}H_{34}O_7$	n.d.	428.2633 $[M+NH_4]^+$	n.d.	2.39	n.d.	428.2641, 369.2262, 309.1689, **249.1473**, 231.1371, 219.1367, 203.1418, 185.1316, 157.1002	n.d.	[45]	*In silico*
168	C1	16.51	**Rudbeckolide** (9-acetoxy-3-methyl-2-oxodec-ahydroazuleno[4,5-b]furan-6,9a(4H)-diyl)bis(methylene) diacetate	Sesquiterpene lactones	$C_{21}H_{30}O_8$	n.d.	428.2272 $[M+NH_4]^+$	n.d.	1.69	n.d.	428.2266, 411.2003, 369.1895, 351.1791, 309.1690, 291.1584, 263.1641, 249.1477, **231.1373**, 185.1317, 157.1005	295	[17]	*In silico*
169	C1	16.54	8-*O*-(4-Hydroxy-3-methyl-butanoyl)salonitenolide	Sesquiterpene lactones	$C_{20}H_{28}O_6$	n.d.	365.1952	n.d.	1.83	n.d.	**365.1953**, 323.1839, 277.1799, 249.1478, 231.1374, 203.1426, 185.1322, 157.1006	n.d.	[46]	*In silico*
171	C1	16.95	Methylbutenyl-hydroxycon-fertin	Sesquiterpene lactones	$C_{20}H_{28}O_6$	n.d.	382.2219 $[M+NH_4]^+$	n.d.	1.41	n.d.	382.2219, 365.1955, 347.1859, 265.1426, **247.1330**, 219.1384, 201.1269	n.d.		*In silico*
177	C1	17.53	Acetyl-butyryl rudbeckin A isomer I	Sesquiterpene lactones	$C_{21}H_{32}O_7$	n.d.	414.2481 $[M+NH_4]^+$	n.d.	1.33	n.d.	397.2212, 379.2119, 291.1955, **249.1482**, 231.1378, 219.1373, 203.1425, 185.1317, 157.0998	n.d.		

179	C1	17.8	Acetyl-butyryl rudbeckin A isomer II	Sesquiterpene lactones	$C_{21}H_{32}O_7$	n.d.	414.2480 $[M+NH_4]^+$	n.d.	1.33	n.d.	397.2222, 379.2108, 291.1944, **249.1481**, 231.1370, 219.1372, 203.1432, 185.1323, 157.1011	n.d.		
181	C1	17.92	Diacetyl-dihydrorudmollin isomer I	Sesquiterpene lactones	$C_{19}H_{28}O_6$	n.d.	370.2214 $[M+NH_4]^+$	n.d.	2.6	n.d.	353.1960, 293.1746, 251.1633, **233.1528**, 187.1473, 159.1166	n.d.	[47]	*In silico*
182	C1	18.07	Acetyl-methylbutenyl dehydrorudbeckin A isomer I	Sesquiterpene lactones	$C_{22}H_{30}O_7$	n.d.	424.2320 $[M+NH_4]^+$	n.d.	2.41	n.d.	424.2333, 407.2077, 307.1523, **265.1427**, 247.1322, 229.1222, 201.1272, 173.0964	n.d.	[48]	
184	C1	18.45	Acetyl-methylbutenoyl rudbeckin A isomer I	Sesquiterpene lactones	$C_{22}H_{32}O_7$	n.d.	426.2479 $[M+NH_4]^+$	n.d.	1.79	n.d.	**409.2217**, 309.1678, 249.1479, 231.1374, 219.1371, 203.1422, 185.1310, 157.1006	*n.d.*	[49]	*In silico*
186	C1	18.64	Acetyl-methylbutenyl dehydrorudbeckin A isomer II	Sesquiterpene lactones	$C_{22}H_{30}O_7$	n.d.	424.2329 $[M+NH_4]^+$	n.d.	2.41	n.d.	424.2327, 407.2046, 347.1877, 307.1526, **265.1432**, 247.1321, 229.1220, 201.1273, 183.1178	n.d.	[48]	*In silico*
188	C1	18.73	Acetyl-butyryl rudbeckin A isomer III	Sesquiterpene lactones	$C_{21}H_{32}O_7$	n.d.	414.2480 $[M+NH_4]^+$	n.d.	1.59	n.d.	397.2216, 379.2107, **249.1486**, 231.1367, 219.1370, 203.1424, 185.1323, 157.1002	n.d.		*In silico*

189	C1	18.87	Diacetyl-propionyl rudbeckin A	Sesquiterpene lactones	$C_{22}H_{32}O_8$	n.d.	442.2435 [M+NH4]+	n.d.	0.1	n.d.	442.2437, 425.2166, 383.2058, 365.1955, **351.1795**, 309.1696, 249.1481, 231.1374, 219.1359, 213.1274, 203.1425, 185.1316, 157.1009	n.d.		*In silico*
196	C1	19.14	Methylbutenyl-confertin A	Sesquiterpene lactones	$C_{20}H_{26}O_5$	n.d.	364.2115 [M+NH4]+	n.d.	1.01	n.d.	364.2132, 347.1862, **247.1322,** 219.1388, 201.1268		[50]	*In silico*
201	C1	19.61	Acetyl-methylbutanoyl rudbeckin A isomer I	Sesquiterpene lactones	$C_{22}H_{34}O_7$	n.d.	428.2643 [M+NH4]+	n.d.	-0.05	n.d.	428.2642, 411.2374, 393.2270, 309.1692, **249.1482**, 231.1375, 219.1375, 203.1425, 185.1321, 157.1011	n.d.	[45]	*In silico*
202	C1	19.73	Methylbutyryl-confertin A	Sesquiterpene lactones	$C_{20}H_{28}O_5$	n.d.	366.2274 [M+NH4]+	n.d.	0.29	n.d.	366.2266, 349.2008, 265.1423, **247.1329,** 235.1300, 219.1380, 191.1064, 173.0952	n.d.		*In silico*
203	C1	19.89	Acetyl-methylbutanoyl rudbeckin A isomer II	Sesquiterpene lactones	$C_{22}H_{34}O_7$	n.d.	428.2639 [M+NH4]+	n.d.	0.92	n.d.	428.2641, 411.2375, 393.2276, 309.1702, **249.1482**, 231.1376,	n.d.	[45]	*In silico*

											219.1374, 203.1429, 185.1315, 157.1008			
206	C1	20.37	Diacetyl-butenoyl rudbeckin A	Sesquiterpene lactones	$C_{23}H_{32}O_8$	n.d.	454.2436 $[M+NH_4]^+$	n.d.	-0.13	n.d.	454.2432, 437.2178, 395.2063, 351.1795, 249.1487, **231.1375**, 213.1268, 185.1319, 157.1005	n.d.	[51]	*In silico*
207	C1	20.56	Acetyl-methylbutenoyl rud-beckin A isomer II	Sesquiterpene lactones	$C_{22}H_{32}O_7$	n.d.	426.2480 $[M+NH_4]^+$	n.d.	1.54	n.d.	409.2189, 309.1688, 267.1574, **249.1491**, 231.1383, 219.1381, 203.1426, 157.1020	n.d.		*In silico*
209	C1	20.71	Acetyl-methylbutaboly rud-beckin A isomer III	Sesquiterpene lactones	$C_{22}H_{34}O_7$	n.d.	428.2643 $[M+NH_4]^+$	n.d.	-0.05	n.d.	411.2380, 393.2277, 309.1685, 267.1582, **249.1486**, 231.1380, 219.1374, 203.1431, 185.1322, 157.1011	n.d.	[45]	*In silico*
210	C1	20.92	Diacetyl-butyryl rudbeckin A (isomer I)	Sesquiterpene lactones	$C_{23}H_{34}O_8$	n.d.	456.2599 $[M+NH_4]^+$	n.d.	-1.61	n.d.	456.2599, 439.2332, 397.2232, 379.2120, **351.1804**, 309.1699, 249.1488, 231.1379, 213.1269, 185.1325, 157.1017	n.d.		*In silico*
212	C1	21.05	Diacetyl-butyryl rudbeckin A (isomer II)	Sesquiterpene lactones	$C_{23}H_{34}O_8$	n.d.	456.2593 $[M+NH_4]^+$	n.d.	-0.24	n.d.	456.2599, 439.2330, 397.2228, 379.2120, **351.1802**, 309.1696, 249.1484, 231.1379,	n.d.		*In silico*

											213.1275, 203.1435, 185.1322, 157.1019			
213	C1	21.27	Methylbutenyl-rudmollin isomer I	Sesquiterpene lactones	$C_{20}H_{28}O_5$	n.d.	366.2278 $[M+NH_4]^+$	n.d.	-0.86	n.d.	366.2280, 349.2014, **249.1484**, 231.1391, 203.1426, 173.0961	n.d.	[52]	*In silico*
215	C1	21.47	Propionyl-methylbutenyl dehydrorudbeckin A	Sesquiterpene lactones	$C_{23}H_{32}O_7$	n.d.	438.2487 $[M+NH_4]^+$	n.d.	-0.17	n.d.	438.2499, 421.2238, 321.1698, **265.1436,** 247.1338, 229.1233, 201.1276	n.d.	[53]	*In silico*
216	C1	21.65	Diacetyl-methylbutenoyl rudbeckin A isomer I	Sesquiterpene lactones	$C_{24}H_{34}O_8$	n.d.	468.2598 $[M+NH_4]^+$	n.d.	-1.35	n.d.	468.2588, 451.2328, 409.2222, **351.1808**, 291.1585, 263.1652, 249.1484, 231.1380, 185.1326, 157.1016	n.d.	[54]	*In silico*
220	C1	21.89	Methylbutenyl-rudmollin isomer II	Sesquiterpene lactones	$C_{20}H_{28}O_5$	n.d.	349.2018	n.d.	-2.44	n.d.	349.2012, **249.1488**, 231.1381, 203.1431, 173.0961	n.d.	[52]	*In silico*
221	C1	21.96	Diacetyl-methylbutenoyl rudbeckin A isomer II	Sesquiterpene lactones	$C_{24}H_{34}O_8$	n.d.	468.2600 $[M+NH_4]^+$	n.d.	-1.79	n.d.	468.2606, 451.2328, 409.2224, **351.1811**, 309.1692, 249.1494, 231.1383, 185.1326	n.d.	[54]	*In silico*
222	C1	22.17	Methylbutyryl-rudmollin	Sesquiterpene lactones	$C_{20}H_{30}O_5$	n.d.	368.2437 $[M+NH_4]^+$	n.d.	-1.57	n.d.	368.2431, 351.2170, **249.1489**, 231.1380, 203.1437, 173.0960	n.d.		*In silico*
223	C1	22.33	Diacetyl-methylbutenoyl rudbeckin A isomer III	Sesquiterpene lactones	$C_{24}H_{34}O_8$	n.d.	468.2602 $[M+NH_4]^+$	n.d.	-2.23	n.d.	468.2593, 451.2328, 409.2221, **351.1802**,	n.d.	[54]	*In silico*

											309.1704, 291.1587, 249.1488, 231.1381, 185.1328			
226	C1	22.65	Diacetyl-methylbutanoyl rudbeckin A (isomer I)	Sesquiterpene lactones	$C_{24}H_{36}O_8$	n.d.	470.2759 $[M+NH_4]^+$	n.d.	-2.34	n.d.	470.2753, 453.2495, 411.2382, **351.1808**, 309.1702, 249.1489, 231.1383, 157.1016	n.d.		*In silico*
228	C1	22.83	Diacetyl-methylbutanoyl rudbeckin A (isomer II)	Sesquiterpene lactones	$C_{24}H_{36}O_8$	n.d.	470.2759 $[M+NH_4]^+$	n.d.	0.62	n.d.	470.2756, 453.2490, 411.2380, 393.2275, **351.1809**, 309.1699, 291.1592, 249.1490, 231.1380, 185.1325, 157.1015	n.d.		*In silico*
239	C1	23.91	Acetyl-methylbutenoyl-propionyl rudbeckin A	Sesquiterpene lactones	$C_{25}H_{36}O_8$	n.d.	482.2748 $[M+NH_4]^+$	n.d.	0.09	n.d.	482.2756, 465.2449, 409.2224, **365.1952**, 309.1687, 291.1587, 249.1478, 231.1378, 185.1316, 157.0997	n.d.	[55]	*In silico*
241	C1	24.11	8α-Tigloyloxy-11β*H*,13-dihydroparthenolide isomer III	Sesquiterpene lactones	$C_{20}H_{28}O_5$	n.d.	349.2006	n.d.	1.01	n.d.	349.2006, 249.1477, **231.1382**, 203.1427, 185.1322, 175.1480, 157.1012	n.d.	[52]	
249	C1	24.86	Acetyl-methylbutenyl-dihydrorudmollin	Sesquiterpene lactones	$C_{22}H_{32}O_6$	n.d.	410.2533 $[M+NH_4]^+$	n.d.	1.06	n.d.	410.2542, 393.2279, 351.2161, **293.1744**, 251.1645, 233.1538, 187.1476, 159.1161	n.d.	[48]	

34	Other	C1	4.13	Cynaroside A (Cynaratriol hexoside)	Sesquiterpenoids	$C_{21}H_{32}O_{10}$	489.1977 $[M+FA-H]^-$	462.2330 $[M+NH_4]^+$	0.11	0.84	281.1392, 251.1289, 237.1496, 219.1365, **207.1386**, 189.1307, 175.1139, 113.0225	462.2327, 283.1534, 265.1415, **253.1429**, 247.1320, 235.1317, 219.1368, 207.1380, 189.1267, 145.1002	n.d.	[56]	
71		Other	6.97	Pulchellamine C	Sesquiterpenoids	$C_{24}H_{33}NO_9$	n.d.	480.2227	n.d.		n.d.	480.2234, 401.1627, **209.0803**, 181.0854	n.d.		*Pulchellamine C (in silico)*
72		C1	7.11	4- OR 15-acetoxyrudmollin	Sesquiterpenoids	$C_{17}H_{24}O_5$	n.d.	309.1691	n.d.	1.79	n.d.	309.1688, **249.1478**, 203.1424, 185.1320, 175.1469	n.d.		*in silico*
98	Other	Other	9.39	Arbusculin E	Sesquiterpenoids	$C_{15}H_{24}O_4$	267.1595	233.1535 $[M-2{\times}H_2O+H]+$	2.17	0.4	**267.1597**, 223.1701	233.1537, 215.1430, 197.1336, **187.1480**, 177.0907, 161.1323, 151.0762, 133.0652	n.d.		*In silico*
130		C2	12.9	Godotol B	Sesquiterpenoids	$C_{15}H_{24}O_2$	n.d.	237.1846	n.d.	1.3	n.d.	**237.1854**, 219.1750, 201.1636, 179.1053, 161.1318, 135.1155	n.d.		*In silico*
149		C1	14.69	4- OR 15-acetoxyrudmollin	Sesquiterpenoids	$C_{17}H_{24}O_5$	n.d.	326.1953 $[M+NH_4]^+$	n.d.	2.92	n.d.	309.1700, **249.1479**, 231.1368, 203.1420, 173.0958	n.d.		*in silico*
150		C2	14.74	(Iso)Kobusone isomer I	Sesquiterpenoids	$C_{14}H_{22}O_2$	n.d.	223.1684	n.d.	3.86	n.d.	**223.1692**, 205.1582, 161.1319, 147.1163, 121.1009, 109.0999	n.d.		*in silico*
154		C2	15.25	(Iso)Kobusone isomer II	Sesquiterpenoids	$C_{14}H_{22}O_2$	n.d.	223.1682	n.d.	4.76	n.d.	**223.1684**, 205.1577, 161.1058, 149.0944	n.d.		*in silico*

192	C1	19.02	3-(acetyloxy)-6-hydroxy-3,6,9-trimethyl-2-oxo-2H,3H,3aH,4H,5H,6H,6aH,7H,9aH,9bH-azuleno[4,5-b]furan-4-yl 2-methylbutanoate OR Epiarguticinin	Sesquiterpenoids	$C_{22}H_{32}O_7$	n.d.	426.2486 $[M+NH_4]^+$	n.d.	0.07	n.d.	409.2221, 351.1796, 309.1689, **249.1478**, 231.1375, 219.1375, 203.1425, 185.1318, 157.1008	*n.d.*	*In silico*
197	C2	19.19	2-[(1S,2S,4aR,8aS)-1-hydroxy-4a-methyl-8-methylidene-1,2,3,4,5,6,7,8a-octahydronaphthalen-2-yl]prop-2-enoic acid	Sesquiterpenoids	$C_{15}H_{22}O_3$	n.d.	251.164	n.d.	0.68	n.d.	**233.1532**, 215.1426, 187.1480, 161.1322	n.d.	*2-[(1S,2S,4aR,8aS)-1-hydroxy-4a-methyl-8-methylidene-1,2,3,4,5,6,7,8a-octahydronaphthalen-2-yl]prop-2-enoic acid*
198	C1	19.37	Diacetyl-dihydrorudmollin isomer II	Sesquiterpenoids	$C_{19}H_{28}O_6$	n.d.	370.2222 $[M+NH_4]^+$	n.d.	0.61	**n.d.**	370.2218, 353.1946, 311.1861, **293.1749**, 251.1632, 233.1534, 205.1581, 187.1474, 159.1161	n.d.	*In silico*
200	C1	19.49	Acetyl-methylbutyryl dehydrorudbeckin A	Sesquiterpenoids	$C_{22}H_{32}O_7$	n.d.	426.2484 $[M+NH_4]^+$	n.d.	0.56	n.d.	426.2480, 409.2201, 365.1932, 307.1531, 265.1434, 247.1327, 229.1219, 201.1280, 173.0973	n.d.	*In silico*
204	Other	20.04	9β-Hydroxyageraphorone isomer	Sesquiterpenoids	$C_{15}H_{24}O_2$	n.d.	254.2111 $[M+NH_4]^+$	n.d.	1.51	n.d.	235.1694, **219.1739**, 201.1634, 175.1474, 137.1316, 123.1169	n.d.	*In silico*
218	C7	21.72	α-Cyperone	Sesquiterpenoids	$C_{15}H_{22}O$	n.d.	219.1743	n.d.	0.19	n.d.	**219.1744**, 201.1638, 159.1169, 145.1014, 119.0856	n.d.	*alpha-Cyperone*

219		C2	21.86	11,13-Dihydrocostunolide	Sesquiterpenoids	$C_{15}H_{22}O_2$	n.d.	235.1695	n.d.	-1.04	n.d.	**235.1688**, 217.1585, 199.1481, 189.1639, 175.1482, 157.1020, 123.1169	n.d.	[57]	Walleminone
224		Other	22.39	13-nor-Eudesm-4,6-dien-11-one	Sesquiterpenoids	$C_{14}H_{20}O$	n.d.	205.1589	n.d.		n.d.	**205.1586**, 187.1477, 162.1041, 149.0960, 135.0806	n.d.		*In silico*
225		C7	22.5	6-hydroxy-Caryophyllene OR Cedr-8(15)-en-9-ol	Sesquiterpenoids	$C_{15}H_{24}O$	n.d.	221.1902	n.d.	-0.95	n.d.	221.1907, **203.1800**, 163.1498, 147.1168, 123.1172	n.d.		*In silico*
232		C1	23.21	Methylbutyryl-dihydrorud-mollin	Sesquiterpenoids	$C_{20}H_{32}O_5$	n.d.	353.2322	n.d.	0.14	n.d.	335.2229, 279.1944, 251.1647, **233.1536**, 221.1535, 205.1583, 187.1484	n.d.		*In silico*
252		C1	25.04	Methylbutyryll-methyl-butenyl dehydrorudbeckin A	Sesquiterpenoids	$C_{25}H_{36}O_7$	n.d.	466.2795 $[M+NH_4]^+$	n.d.	0.96	n.d.	**466.2796**, 449.2528, 365.1972, 349.2005, 265.1439, 247.1328, 219.1374, 201.1261, 173.0966	n.d.		
31	Other	Other	3.88	*p*-Menthane-triol hexoside isomer I	Terpene glycosides	$C_{16}H_{30}O_8$	395.1924 $[M+FA-H]^-$	351.2008	-0.37	**1.55**	**349.1883**, 187.1333	315.1782, **153.1269**, 145.0485, 135.1168	n.d.		*Hexose + C10H19O2*
38	Other	C8	4.36	*p*-Menthane-triol hexoside isomer II	Terpene glycosides	$C_{16}H_{30}O_8$	395.1919 $[M+FA-H]^-$	333.1905 $[M-H_2O+H]^+$	1.06	0.8	187.1328	315.1800, 243.1379, **153.1265**	n.d.		*Hexose + C10H19O2*
51	Other		5.12	Sachaloside II OR Sacranoside A isomer	Terpene glycosides	$C_{21}H_{34}O_{10}$	491.213 [M+FA-H]-		0.9		283.1532,		n.d.	[58]	*in silico*

92	Other	8.92	Dimethyloctadiendiol hexoside	Terpene glycosides	$C_{16}H_{28}O_7$	377.1809 $[M+FA-H]^-$	350.2170 $[M+NH_4]^+$	2.43	0.99	n.d.	**153.1274**, 147.0439, 135.1159	n.d		*NCGC00385387-01_C16H28O7_4-(Hydroxymethyl)-1-isopropyl-3-cyclohexen-1-yl beta-D-glucopyranoside*
49	C1	5.08	Minwanensin	Terpene lactones	$C_{15}H_{24}O_5$	n.d.	285.1688	n.d.	2.99	n.d.	267.1592, **249.1480**, 231.1376, 219.1374, 203.1428, 185.1321	n.d.	[59]	
230	Other	23.07	Cashmeran	Terpenoids	$C_{14}H_{22}O$	n.d.	207.1744	n.d.	-0.28	n.d.	**207.1745**, 189.1640, 151.1122, 123.0811	n.d.		*Cashmeran*

Table S2. Relative %CAD area of groups of compounds found in *R. hirta*.

Compound class	% of total CAD area
Fatty acyl glycosides	0.0%
O-glucuronides	0.1%
Glycosylmonoacylglycerols	0.2%
1-acyl-*sn*-glycero-3-phosphoethanolamines	0.2%
Hydroxycinnamic acids	0.2%
Diterpene glycosides	0.4%
Eudesmane glycosides	0.5%
Terpene glycosides	0.6%
Biflavonoids and polyflavonoids	1.5%
Hydroxycinnamic acid glycosides	2.1%
Sesquiterpenoids	3.7%
Fatty acids	5.9%
Coumaric acids and derivatives	7.8%
Amino acids and derivatives	8.6%

Quinic acids and derivatives	19.1% (quinic acid = 12.4%, other = 6.7%)
Flavonoid O-glycosides	23.2%
Sesquiterpene lactones	26.0%
SUM	*100%*
